# Prefrontal Cortical Regulation of REM Sleep

**DOI:** 10.21203/rs.3.rs-1417511/v1

**Published:** 2023-10-10

**Authors:** Franz Weber, Jiso Hong, David Lozano, Kevin Beier, Shinjae Chung

**Affiliations:** University of Pennsylvania; University of Pennsylvania; University of Pennsylvania; University of California, Irvine; University of Pennsylvania

## Abstract

Rapid-eye-movement (REM) sleep is accompanied by intense cortical activity, underlying its wake-like electroencephalogram (EEG). The neural activity inducing REM sleep is thought to originate from subcortical circuits in brainstem and hypothalamus. However, whether cortical neurons can also trigger REM sleep has remained unknown. Here, we show in mice that the medial prefrontal cortex (mPFC) strongly promotes REM sleep. Bidirectional optogenetic manipulations demonstrate that excitatory mPFC neurons promote REM sleep through their projections to the lateral hypothalamus (LH) and regulate phasic events, reflected in accelerated EEG theta oscillations and increased eye-movement density during REM sleep. Calcium imaging reveals that the majority of LH-projecting mPFC neurons are maximally activated during REM sleep and a subpopulation is recruited during phasic theta accelerations. Our results delineate a cortico-hypothalamic circuit for the top-down control of REM sleep and identify a critical role of the mPFC in regulating phasic events during REM sleep.

## INTRODUCTION

Seminal transection studies by Jouvet localized the circuits necessary for the generation of rapid-eye-movement (REM) sleep within the brainstem^1^. The commonly held view is that REM sleep is initiated by subcortical circuits in brainstem and hypothalamus, causing an intense activation of the forebrain including cortical areas, which in turn gives rise to its characteristic wake-like electroencephalogram (EEG)^2–5^. Within the cortex, the unique neurophysiological state of REM sleep plays important roles in mnemonic processing and synaptic plasticity^6,7^. In particular, phasic events during REM sleep, which are characterized by rapid eye movements (EMs) and reflected in intensified hippocampal theta (θ) oscillations, provide windows of opportunity for inter-regional coordination between hippocampal and cortical regions and have therefore been implicated in memory processing^8–11^. Dysregulation of phasic REM sleep is known to be associated with psychiatric disorders such as major depressive disorder (MDD), which are accompanied by cortical dysfunction^12,13^. However, despite REM sleep’s functional importance for cortex, the mechanisms by which cortical neurons may regulate REM sleep and its phasic activity have largely remained unknown.

The medial prefrontal cortex (mPFC) is known to interact with multiple brain regions through its long-range connections, allowing it to coordinate various behaviors ranging from emotional processing to the regulation of the autonomic nervous system^14–17^ and to control brain-wide activity dynamics^18^. Downstream regions innervated by the mPFC include subcortical areas in brainstem and hypothalamus involved in sleep-wake regulation. Functional imaging studies in humans demonstrated that the ventromedial PFC (vmPFC, homolog of the rodent mPFC) is strongly activated during REM sleep^2,5^. Yet whether neural activity in this prefrontal area is directly involved in REM sleep regulation remains unclear. Using bidirectional optogenetic manipulation and *in vivo* calcium imaging in mice, we show that excitatory neurons in the mPFC promote REM sleep through their projections to the lateral hypothalamus (LH) and enhance phasic REM sleep, reflected in transiently intensified θ oscillations in the EEG and an increased EM density. These results establish a prominent role of the mPFC in REM sleep regulation and may therefore provide a mechanistic explanation for the close association between psychiatric disorders and disturbances in REM sleep.

## RESULTS

### Optogenetic activation of mPFC Pyr neurons induces REM sleep

To monitor the activity of excitatory pyramidal (Pyr) neurons in the mPFC during spontaneous sleep, we expressed GCaMP6s in the mPFC of C57BL/6J mice under the control of the CaMKII promoter and performed fiber photometry imaging via optic fibers implanted into the infralimbic (IL) cortex (**Extended Data Fig. 1a,b;** Methods). The population calcium activity of mPFC Pyr neurons was highest during REM sleep, followed by wake and NREM sleep (**Extended Data Fig. 1c,d**). Their activity started increasing before the transition to REM sleep and remained elevated throughout REM sleep (**Extended Data Fig. 1e;** Methods).

**Figure 1:**
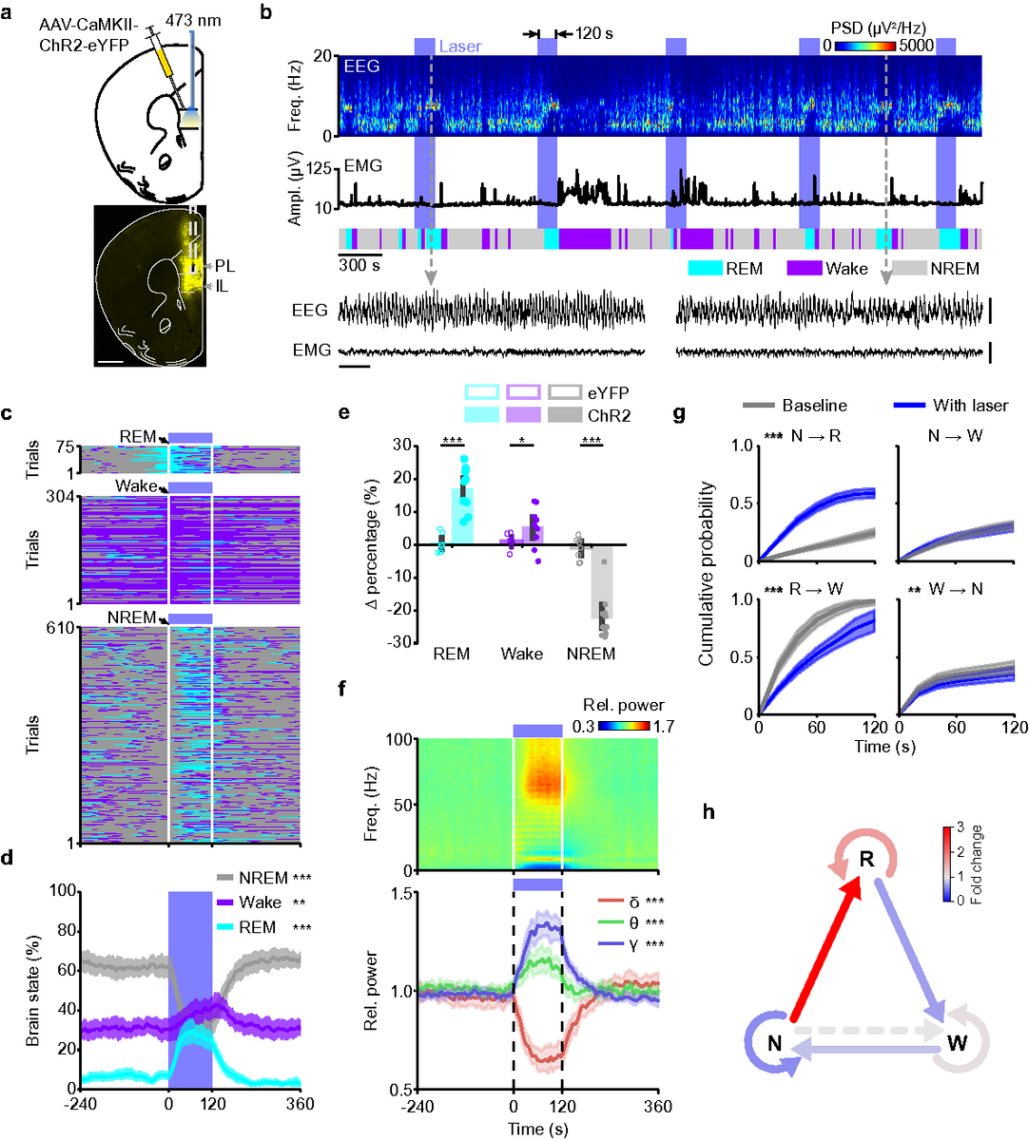
Optogenetic activation of mPFC Pyr neurons triggers REMs. (**a**) Top, schematic of optogenetic activation of mPFC Pyr neurons. Bottom, expression of AAV-CaMKII-ChR2-eYFP (yellow) in mPFC of a C57BL/6J mouse. Dashed lines, optic fiber tract. Scale bar, 500 μm. IL, infralimbic cortex; PL, prelimbic cortex. Brain atlas image adapted with permission from ref.^61^. (**b**) Example open-loop stimulation experiment. Shown are EEG spectrogram, EMG amplitude, brain states, and EEG, EMG raw traces at an expanded timescale for two time points (gray lines); scale bars, 1 s and 0.5 mV. Blue patches, 120 s laser stimulation intervals (473 nm, 5 Hz). PSD, power spectral density. (**c**) Brain states in all stimulation trials from n = 11 mice aligned by the laser onset at t = 0 s. Trials were sorted depending on the brain state at laser onset (arrows). (**d**) Percentages of brain states before, during, and after open-loop stimulation. Blue patch, laser stimulation interval. Two-way repeated-measures (rm) ANOVA comparing the mean percentage of each brain state between the laser and preceding 120 s baseline interval (interaction, P = 0.0000); t-tests with Holm-Bonferroni correction; baseline vs laser for REM, P = 0.0000; for wake, P = 0.0093; for NREM, P =0.0000. n = 11 mice. Lines, averages across mice; shadings, 95% confidence intervals (CIs). (**e**) Changes in the percentage of each brain state (difference between preceding 120 s baseline and laser interval) induced by laser stimulation in ChR2 and eYFP mice. Mixed ANOVA with brain state as within and virus as between factor (interaction, P = 0.0000); t-tests with Holm-Bonferroni correction; eYFP vs ChR2 for REM, P = 0.0000; for wake, P = 0.0321; for NREM, P = 0.0000. ChR2, n = 11; eYFP, n = 8 mice. Bars, averages across mice; dots, individual mice; error bars, 95% CIs. (**f**) Top, laser-trial averaged EEG spectrogram (normalized by the mean power in each frequency band; Methods). Bottom, time course of δ (0.5 – 4.5 Hz), θ (6 – 9.5 Hz), and γ power (50 – 90 Hz) before, during, and after laser stimulation. Two-way rm ANOVA comparing the mean power in each frequency band between the laser and preceding 120 s baseline interval (interaction, P = 0.0000); t-tests with Holm-Bonferroni correction; baseline vs laser: δ, P = 0.0000; θ, P = 0.0006; γ, P = 0.0000. n = 11 mice. Lines, averages across mice; shadings, 95% CIs. (**g**) Cumulative probabilities to transition from brain state X at laser onset (t = 0 s) to state Y within the laser interval (blue) and the 120 s baseline interval preceding laser onset (gray). Bootstrap; N→R, P = 0.0001; R→W, P = 0.0001; N→W, P = 0.9274; W→N, P = 0.0042; n = 11 mice. Shadings, 95% CIs. (**h**) Graph summarizing relative changes in the cumulative transition probabilities between baseline and laser interval (Methods). A value of 1 indicates no change between baseline and laser. The edges for Wake→REM and REM→NREM transitions were omitted, as these types of transitions were not observed in the dataset. Solid and dashed lines indicate significant and non- significant changes in the transition probabilities, respectively. See **Supplementary Table 1** for detailed statistical information. *P<0.05, **P<0.01, ***P<0.001.

To test whether activation of the mPFC is causally involved in REM sleep regulation, we expressed adeno-associated viruses (AAVs) encoding ChR2-eYFP in mPFC Pyr neurons for optogenetic excitation (Fig. 1a; Methods). Within the mPFC, ChR2-eYFP was mainly expressed in the IL and prelimbic (PL) cortex, and the optic fibers for laser stimulation were placed on top of the IL (**Extended Data Fig. 2a**). Fluorescence in situ hybridization (FISH) confirmed the specificity of ChR2-eYFP expression in excitatory mPFC neurons (**Supplementary Fig. 1a,c**). Using an open-loop stimulation protocol, we randomly applied laser stimulation every 13 – 17 min for 120 s (473 nm, 5 Hz), while recording EEG and electromyogram (EMG) signals to classify the animal’s brain state (Fig. 1b; Methods). Surprisingly, we frequently observed that laser stimulation coincided with REM sleep, particularly when the laser onset fell on NREM sleep (Fig. 1b,c; **Supplementary Video 1**). The average latency between the laser onset and the onset of a REM sleep episode induced during the 120 s laser interval was 41.13 s (95% confidence interval (CI) [37.50 s, 45.19 s]). To quantify the effect of mPFC activation on the brain state, we aligned the laser trials from all mice by the onset of laser stimulation (at t = 0 s; Fig. 1c) and calculated for each time point the percentage of trials that the mice spent in wake, non-REM (NREM), or REM sleep (Fig. 1d). We found that optogenetic activation of mPFC Pyr neurons induced a marked increase in the percentage of REM sleep during laser stimulation and a complementary decrease in NREM sleep (Fig. 1d,e). In control mice expressing eYFP, laser stimulation had no effect (**Extended Data Fig. 2b,c**) and the laser-induced changes in the brain states consequently differed between ChR2 and eYFP mice (Fig. 1e).

Consistent with the REM sleep-facilitating effect of mPFC Pyr neuron activation, the laser-trial averaged EEG spectrogram exhibited a strong increase in the θ (6.0 – 9.5 Hz) and gamma range (γ, 50 – 90 Hz), both of which are prominent during REM sleep, with a concomitant reduction in the delta (δ, 0.5 – 4.5 Hz) power (Fig. 1f). Compared to the EEG in eYFP mice, activation of mPFC Pyr neurons during REM sleep caused a small increase in the θ and sigma (σ, 10 – 15 Hz) power (**Extended Data Fig. 2f**), and mPFC stimulation attenuated the δ power in all brain states (**Extended Data Fig. 2f–h**). Optogenetic activation did not alter the EMG amplitude in any brain state, and there were no specificity differences in the laser-induced changes between ChR2 and eYFP mice (**Extended Data Fig. 2i**).

The increase in REM sleep during the laser interval was matched by a reduction in NREM sleep (Fig. 1d,e), indicating that this effect is primarily the result of an increase in NREM→REM transitions. For direct quantification, we calculated the cumulative probability that the animal transitions from NREM sleep at laser onset (t = 0 s) to REM sleep within the 120 s laser interval (Fig. 1g; Methods). Activation of mPFC Pyr neurons markedly elevated the cumulative probability of NREM→REM transitions throughout the laser interval compared with that during the preceding 120 s baseline interval without laser (Fig. 1g,h), and the laser-induced changes in the transition probability were significantly different between the ChR2 and eYFP control mice (**Extended Data Fig. 2e**). The strong enhancement of NREM→REM transitions explains the high success rate of triggering REM sleep (57.91%, CI [52.21%, 63.03%]), when the laser onset fell on NREM sleep (Fig. 1c), and consequently reduced the probability to remain in NREM sleep (Fig. 1h, **Extended Data Fig. 2d,e**). In contrast, we did not observe Wake→REM transitions, indicating that the ability of mPFC Pyr neurons to initiate REM sleep fell under the same constraints as the induction of REM sleep during spontaneous sleep, where Wake→REM transitions are rarely observed. Since in rodents REM sleep is typically followed by wake bouts, the reduced REM→Wake transition probability (Fig. 1g,h) resulted in an increased maintenance of REM sleep throughout laser stimulation (Fig. 1g,h, **Extended Data Fig. 2d,e**). Compared with the strong effects on transitions into and out of REM sleep, activation of the mPFC caused a comparably weak reduction in the probability of Wake→NREM transitions in ChR2 mice (Fig. 1g,h), explaining the small increase in the percentage of wake during the laser interval (Fig. 1e). In sum, brain state-dependent analysis of the laser effects revealed that excitation of mPFC Pyr neurons promotes NREM→REM transitions and maintains REM sleep.

### mPFC Pyr neurons maintain REM sleep and promote phasic REM

**Figure 2:**
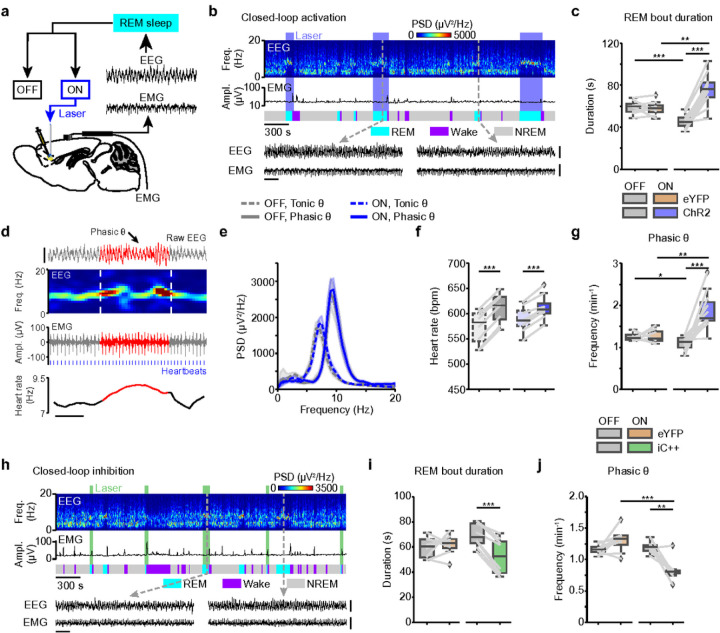
Activation of mPFC Pyr neurons maintains REM sleep and promotes phasic θ events. (**a**) Schematic of optogenetic closed-loop stimulation during REM sleep. Brain atlas image adapted with permission from ref.^61^. (**b**) Example illustrating closed-loop activation of mPFC Pyr neurons during REM sleep (blue patches; 473 nm, 5 Hz). Shown are EEG spectrogram, EMG amplitude, brain states, and EEG, EMG raw traces for two time points (gray lines); scale bars, 1 s and 0.5 mV. (**c**) Duration of REM sleep episodes with (ON) and without closed-loop stimulation (OFF) in mice expressing eYFP or ChR2 in mPFC Pyr neurons. Mixed ANOVA with laser (P = 0.0000) as within and virus as between factor (interaction, P = 0.0000); t-tests with Holm-Bonferroni correction; OFF vs ON: eYFP, P = 0.7370; for ChR2, P = 0.0001; eYFP vs ChR2: OFF, P = 0.0009; ON, P = 0.0046. ChR2, n = 11; eYFP, n = 8 mice. Box plots (see Methods for definition); lines, individual mice. (**d**) Example of a phasic θ event (red). Shown are raw EEG, EEG spectrogram, raw EMG and heartbeats (blue ticks) detected in the EMG (Methods). Scale bars, 1 s and 250 μV. (**e**) PSD of the EEG during phasic θ events and remaining REM sleep (tonic θ) with (ON) and without laser (OFF). The peak frequency and θ power (5 – 12 Hz) were increased during phasic θ events. Peak frequency, two-way rm ANOVA with type of REM (tonic or phasic, P = 0.0000) and laser (OFF or ON) as within factors (interaction, P = 0.0046); t-tests with Holm-Bonferroni correction; tonic vs phasic: OFF, P = 0.0000; ON, P = 0.0000. θ power, two-way rm ANOVA (type, P = 0.0000; interaction, P = 0.8041); t-tests; tonic vs phasic: OFF, P = 0.0000; ON, P = 0.0000. n = 11 mice. Lines, averages across mice; shadings, 95% CIs. (**f**) Heart rate during phasic θ events and remaining REM sleep (tonic θ) with (ON) and without laser (OFF). Two-way rm ANOVA with type of REM (tonic or phasic, P = 0.0000) and laser (OFF or ON) as within factors (interaction, P = 0.0016); t-tests with Holm-Bonferroni correction; tonic vs phasic: OFF, P = 0.0000; ON, P = 0.0000. n = 8 mice. Box plots; lines, individual mice; bpm, beats per minute. (**g**) Frequency of phasic θ events during REM sleep episodes with (ON) and without laser (OFF) in eYFP and ChR2 mice. Mixed ANOVA with laser as within (P = 0.0001) and virus as between factor (interaction, P = 0.0007); pairwise t-tests with Holm-Bonferroni correction; OFF vs ON: eYFP, P = 0.9671; ChR2, P = 0.0007; eYFP vs ChR2: OFF, P = 0.0195; ON, P = 0.0019. ChR2, n = 11; eYFP, n = 8 mice. Box plots; lines, individual mice. (**h**) Example illustrating closed-loop inhibition of mPFC Pyr neurons using iC++ during REM sleep (green patches; 473 nm, step pulse). Scale bars, 1 s and 0.5 mV. (**i**) Duration of REM sleep episodes with and without closed-loop inhibition of mPFC Pyr neurons in eYFP and iC++ mice. mixed ANOVA (laser, P = 0.0032; interaction, P = 0.0004); pairwise t-tests with Holm-Bonferroni correction; OFF vs ON: eYFP, P = 0.5312; iC++, P = 0.0002; eYFP vs iC++: OFF, P = 0.0988; ON, P = 0.1345. iC++, n = 8; eYFP, n = 8 mice. Box plots; lines, individual mice. (**j**) Frequency of phasic θ events during REM sleep episodes with and without closed-loop inhibition in eYFP and iC++ mice. Mixed ANOVA (laser, P = 0.0473; interaction, P = 0.0002); pairwise t-tests with Holm-Bonferroni correction; OFF vs ON: eYFP, P = 0.0703; iC++, P = 0.0039; eYFP vs. iC++: OFF, P = 0.4757; ON, P = 0.0003. iC++, n = 8; eYFP, n = 8 mice. Box plots; lines, individual mice. See **Supplementary Table 1** for detailed statistical information. *P<0.05, **P<0.01, ***P<0.001.

To directly investigate the role of the activity of mPFC Pyr neurons in maintaining REM sleep, we performed closed-loop stimulation experiments, in which laser stimulation was initiated as soon as a spontaneous REM sleep onset was detected and lasted till the end of the REM sleep episode (Fig. 2a,b; **Supplementary Fig. 2;** Methods). The laser was turned on for a randomly selected subset (~50%) of the detected episodes, resulting in a balanced dataset of REM sleep episodes with (laser-on episodes) and without laser stimulation (laser-off episodes). Closed-loop stimulation significantly increased the EEG θ and σ power, while reducing the δ power during REM sleep (**Extended Data Fig. 3a,b**). Consistent with the increased probability to maintain REM sleep found for open-loop stimulation (Fig. 1h, **Extended Data Fig. 2d**), closed-loop activation of mPFC Pyr neurons prolonged the duration of REM sleep episodes compared with the duration of laser-off episodes in the same animals and the duration of laser-on episodes in eYFP controls (Fig. 2c), whereas there was no significant effect in the eYFP controls. The duration of laser-on episodes in ChR2 mice was also significantly longer in comparison with the average duration of REM sleep in baseline recordings without laser, obtained on separate experimental days (**Extended Data Fig. 3c**).

In rodents^8,9,11,19^, cats^10^, and macaques^20^, the persistent θ oscillations in the EEG during REM sleep are interleaved with sudden phasic increases in the θ frequency and power (Fig. 2d). These phasic θ events are accompanied by increased activity in the hippocampus^11^ and associated with rapid EMs^19^, an increased heart rate, and vascular hyperactivity^21,22^. To study the effect of mPFC Pyr activation, we detected phasic θ events during REM sleep from the parietal EEG using an algorithm previously applied in rodents^23^ (Methods). During phasic θ events, the peak frequency and power of the θ oscillations were clearly increased^8,9^ (Fig. 2e) and the heart rate was significantly accelerated for both REM episodes with and without laser (Fig. 2f; see Methods and **Supplementary Fig. 3** for heart rate detection). Interestingly, optogenetic activation of mPFC Pyr neurons increased the frequency of phasic θ events during REM sleep (Fig. 2g). In eYFP control mice, laser stimulation had no effect on phasic θ events, and the effect of laser was significantly different between ChR2 and eYFP mice (Fig. 2g). Correlating the time from the REM sleep onset with the frequency of phasic θ events in baseline recordings without laser stimulation showed that their occurrence becomes less likely the longer REM sleep lasts (**Extended Data Fig. 3j**), excluding the possibility that the increase in their frequency resulted from the extension of the REM bout duration by closed-loop stimulation.

Next, to examine how inactivation of the mPFC affects REM sleep, we expressed the light-activated chloride channel iC++ in mPFC Pyr neurons (**Extended Data Fig. 3d**) and performed closed-loop inhibition (Fig. 2h). Using FISH, we confirmed the specificity of iC++-eYPF expression (**Supplementary Fig. 1b,c**). Silencing mPFC Pyr neurons attenuated the θ and σ power in the EEG during REM sleep (**Extended Data Fig. 3e,f**). In contrast to ChR2 mice, closed-loop inhibition shortened REM sleep episodes in iC++ mice (Fig. 2i), and the decrease in the REM sleep duration was also significant when compared with the duration in baseline recordings without laser stimulation in iC++ mice (**Extended Data Fig. 3g**). Moreover, inactivation of mPFC Pyr neurons reduced the frequency of phasic θ events (Fig. 2j), an effect not observed in eYFP controls. As found for ChR2 mice, in iC++ animals the frequency and power of the θ oscillations as well as the heart rate were also increased during phasic θ events in both laser-on and laser-off episodes (**Extended Data Fig. 3h,i**). In summary, the results from closed-loop manipulation support a role of mPFC Pyr neuron activity in the maintenance of REM sleep and regulation of phasic REM sleep.

Notably, the mean duration of laser-off REM sleep episodes was longer for iC++ than for ChR2 mice (compare Fig. 2c and **2i**). But the mean duration of all REM sleep episodes, including both episodes with and without laser, did not differ between ChR2 and iC++ mice as well as their corresponding eYFP controls (**Extended Data Fig. 3k**). This suggests that a change in the duration of REM sleep episodes caused by mPFC activation or inhibition is compensated by an opposite change in the duration of laser-off episodes, indicating the presence of a homeostatic mechanism that preserves the mean duration of REM sleep. Consistent with this notion, the duration of laser-off episodes in ChR2 mice was shorter than the REM sleep duration in baseline recordings without laser, while the opposite was the case for iC++ mice (**Extended Data Fig. 3c,g**).

Among the behaviorally most salient features of phasic REM sleep are the eponymous rapid EMs. To test whether, in addition to phasic θ events, mPFC activity also regulates the frequency of rapid EMs during REM sleep, we tracked the pupil movement using a camera in head-fifixed animals expressing ChR2 or iC++ in mPFC Pyr neurons (Extended Data Fig. 4a,b)^24,25^. The amount, duration, and frequency of REM sleep in head-fifixed mice was comparable to that of freely moving animals (**Supplementary Fig. 4**). Rapid EMs were identified as sudden accelerations in the pupil speed during REM sleep (Methods**, Supplementary Fig. 5**, and **Supplementary Video 2**), and the distribution of the intervals between rapid EMs showed that these often occur in bufirsts^19^, i.e. sequences of two or more closely interspaced EMs (separated by less than 250 ms; **Extended Data Fig. 4c**). Consistent with the previously reported correlation between EMs and increases in the EEG θ frequency and power^19^, we found that rapid EMs occurred more frequently during phasic θ events than during tonic θ oscillations and the frequency of EM burstswas also strongly elevated (**Extended Data Fig. 4d**).

To test whether excitation of mPFC Pyr neurons alters the frequency of EMs, we performed closed-loop stimulation experiments. As observed for freely moving animals, activation of mPFC Pyr neurons prolonged REM sleep compared with laser-off episodes recorded within the same session and compared with episodes from baseline recordings without laser in the same animals (**Extended Data Fig. 4e**), and laser stimulation also increased the frequency of phasic θ events (**Extended Data Fig. 4f**). Conversely, iC++-mediated inhibition during REM sleep decreased the duration of REM sleep and the frequency of θ events (**Extended Data Fig. 4i,j**). Interestingly, optogenetically exciting the mPFC neurons during REM sleep increased the overall frequency of EMs and the rate of EM bursts(**Extended Data Fig. 4g**), while inactivating these neurons had the opposite effects (**Extended Data Fig. 4k**). The latency to the first EM (from the onset of REM sleep) was not significantly changed by mPFC manipulation (**Extended Data Fig. 4h,l**). To exclude the possibility that the observed changes in the EM density are solely the result of the laser-induced changes in the REM sleep duration, we divided baseline and laser-on REM sleep bouts into different duration bins and compared the EM frequency in each bin. In both ChR2 and iC++ mice, laser stimulation significantly altered the frequency of EMs and EM bursts, and the magnitude of the effect depended on the REM sleep duration (**Extended Data Fig. 4m,n**). For REM sleep durations up to 80 s, for which the baseline frequency was comparably low, mPFC activation strongly enhanced the frequency of EMs and EM bursts (**Extended Data Fig. 4m**). In contrast, the effect of mPFC inhibition was strongest for long REM sleep episodes (> 80 s), for which the baseline frequency was highest (**Extended Data Fig. 4n**). In sum, these findings support a role of mPFC Pyr neuron activity in the regulation of phasic REM sleep, by promoting both phasic θ events and rapid EMs.

### mPFC inhibitory neurons suppress REM sleep

**Figure 3:**
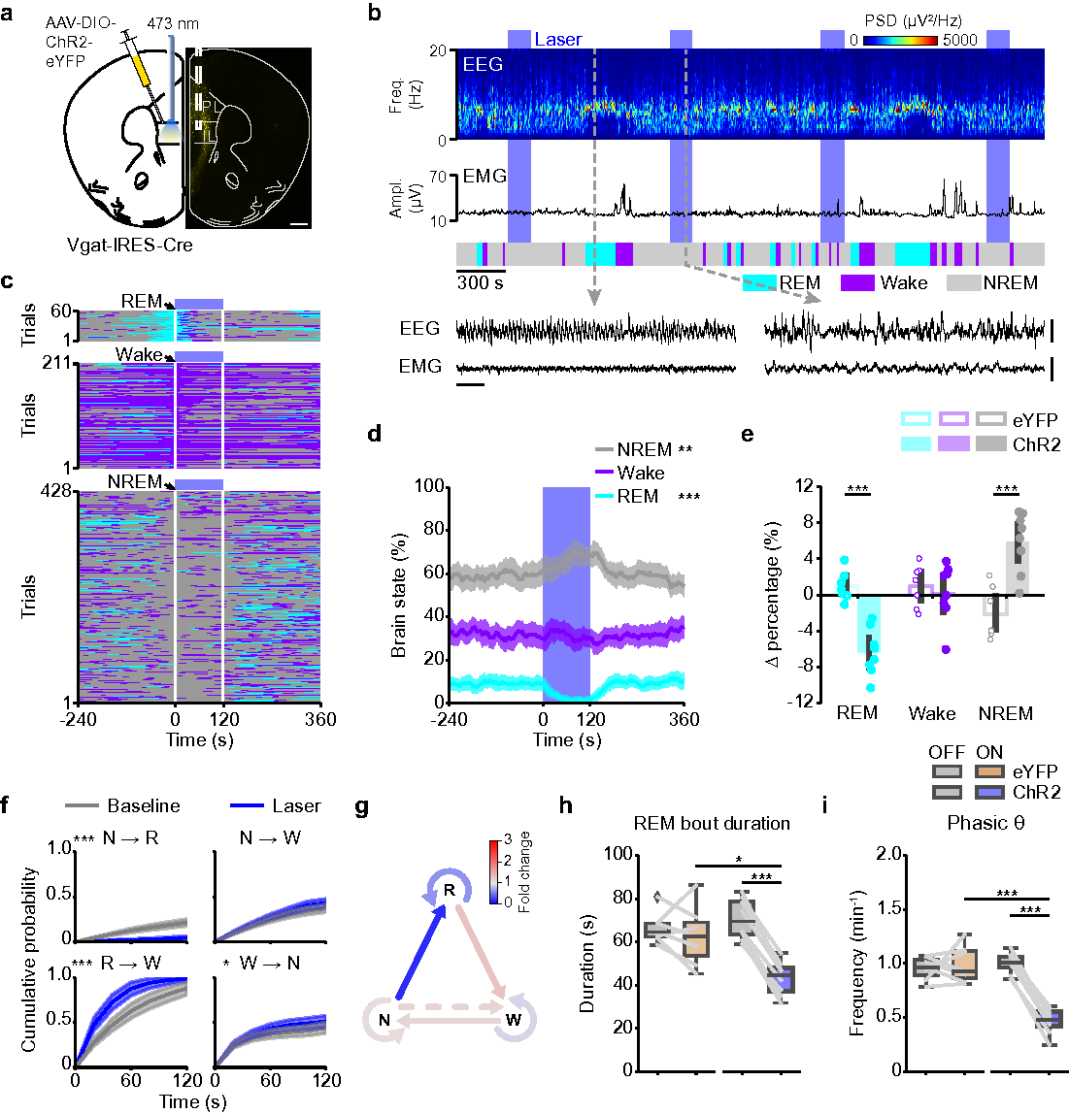
Optogenetic activation of mPFC inhibitory neurons suppresses REM sleep. (**a**) Left, schematic illustrating optogenetic activation of mPFC Vgat neurons. Right, fluorescence image in a Vgat-IRES-Cre mouse injected with AAV-DIO-ChR2-eYFP. Dashed lines, optic fiber tract. Scale bar, 500 μm. Brain atlas image adapted with permission from ref.^61^. (**b**) Example open-loop experiment in a Vgat-IRES-Cre mouse. Shown are EEG spectrogram, EMG amplitude, brain states, and EEG, EMG raw traces for two time points (gray lines); scale bars, 1 s and 0.5 mV. Blue patches, 120 s laser stimulation intervals (473 nm, 20 Hz). (**c**) Laser stimulation trials from all n = 8 mice, sorted depending on the brain state at laser onset (t = 0 s, arrows). (**d**) Percentages of brain states before, during, and after laser stimulation. Blue bar, laser stimulation interval. Two-way rm ANOVA (interaction, P = 0.0001); t-tests with Holm-Bonferroni correction; baseline vs laser: REM, P = 0.0007; wake, P = 0.7588; NREM, P = 0.0029. n = 8 mice. Lines, averages across mice; shadings, 95% CIs. (**e**) Changes in the percentage of each brain state (difference between preceding 120 s baseline and laser interval) induced by laser stimulation in ChR2 and eYFP mice. Mixed ANOVA (interaction, P = 0.0000); t-tests with Holm-Bonferroni correction; eYFP vs ChR2: REM, P = 0.0001; wake, P = 0.6354; NREM, P = 0.0005. ChR2, n = 8; eYFP, n = 7 mice. Bars, averages across mice; dots, individual mice; error bars, 95% CIs. (**f**) Cumulative transition probabilities during baseline (120 s interval before laser onset) and laser interval. Bootstrap; N→R, P = 0.0001; R→W, P = 0.0001; N→W, P = 0.0824; W→N, P = 0.0246; n = 8 mice. Shadings, 95% CIs. (**g**) Graph visualizing the laser-induced changes in the cumulative transition probabilities. (**h**) Duration of REM sleep episodes with (ON) and without closed-loop stimulation (OFF) in eYFP and ChR2 mice. Mixed ANOVA (laser, P = 0.0000; interaction, P = 0.0001); t-tests with Holm-Bonferroni correction; OFF vs ON: eYFP, P = 0.4062; ChR2, P = 0.0000; eYFP vs ChR2: OFF, P = 0.3690; ON, P = 0.0183. ChR2, n = 8; eYFP, n = 7 mice. Box plots; lines, individual mice. (**i**) Frequency of phasic θ events during REM sleep episodes with and without laser in eYFP and ChR2 mice. Mixed ANOVA (laser, P = 0.0000; interaction, P = 0.0000); t-tests with Holm-Bonferroni correction; OFF vs ON: eYFP, P = 0.6309; ChR2, P = 0.0000; eYFP vs ChR2: OFF, P = 0.3836; ON, P = 0.0001. ChR2, n = 8; eYFP, n = 7 mice. Box plots; lines, individual mice. See **Supplementary Table 1** for detailed statistical information. *P<0.05, **P<0.01, ***P<0.001.

To test whether the REM sleep-promoting effect is specific to excitatory mPFC neurons, we activated inhibitory neurons by injecting AAV-DIO-ChR2-eYFP into the mPFC of Vgat-IRES-Cre mice and implanting an optic fiber into the mPFC (Fig. 3a, **Extended Data Fig. 5a**). Open-loop activation of mPFC GABAergic neurons suppressed REM sleep during the laser interval and increased NREM sleep (Fig. 3b–d). In contrast, there was no effect in eYFP control mice(**Extended data Fig. 5b,c**) and the laser-induced changes differed between ChR2 and eYFP animals (Fig. 3e). Laser stimulation strongly reduced the probability of NREM→REM transitions, which led to the reduction of REM sleep and an enhanced maintenance of NREM sleep (Fig. 3f,g, **Extended Data Fig. 5d,e**). Due to an increase in REM→Wake transitions, the maintenance of REM sleep was impaired (Fig. 3f,g, **Extended Data Fig. 5d,e**). Accordingly, closed-loop stimulation of GABAergic neurons significantly shortened the duration of REM sleep episodes compared with laser-off episodes and with laser-on episodes in eYFP control mice (Fig. 3h). In contrast to mPFC Pyr neurons, activation of GABAergic neurons slightly reduced the EEG θ and σ power, while increasing the δ power (**Extended Data Fig. 5f,g**) and strongly decreased the frequency of phasic θ events (Fig. 3i). In summary, activation of mPFC inhibitory neurons suppressed transitions to REM sleep, shortened the duration of REM sleep episodes and reduced phasic θ events.

### mPFC→LH neurons regulate REM sleep and phasic θ events

The mPFC sends long-range projections to multiple brain regions including cortical and subcortical areas^15–17^, but the projections relevant to REM sleep regulation are unknown. To label axonal projections for anterograde tracing, we expressed tdTomato in mPFC Pyr neurons (**Extended Data Fig. 6a,b;** Methods). Consistent with previous studies, we found dense projections to multiple areas, including the basolateral amygdala (BLA), LH, thalamic nuclei (ventral anterior, mediodorsal (MD), and reuniens thalamic nucleus), lateral and dorsolateral periaqueductal gray (PAG) and laterodorsal tegmental nucleus (LDTg) (**Extended Data Fig. 6c–g**). To probe the functional role of different subcortical projections in REM sleep regulation, we injected AAV-CaMKII-ChR2-eYFP into the mPFC and implanted an optic fiber into the MD, LH, PAG, or LDTg, located in thalamus, hypothalamus, midbrain, and brainstem, respectively. Interestingly, among the tested pathways only activation of mPFC projections to the LH (**Extended Data Fig. 7a,b**) significantly enhanced NREM→REM transitions (**Extended Data Fig. 7c,d**), reflected in an increase in the θ and γ power and reduced δ power in the trial averaged EEG spectrogram (**Extended Data Fig. 7e**). Closed-loop stimulation prolonged the duration of REM sleep (**Extended Data Fig. 7f**) and enhanced the frequency of phasic θ events (**Extended Data Fig. 7g**). In contrast, stimulating the projections to the other tested areas did not affect the REM sleep percentage, duration, or phasic θ events (**Extended Data Fig. 7h–v**). Thus, mPFC projections to the LH functionally contribute to both the regulation of REM sleep and phasic θ events.

To specifically label mPFC neurons projecting to the LH (mPFC→LH neurons), we injected AAVrg-Cre, an AAV with high retrograde efficiency^26^, into the LH and observed robust labeling of neurons in the mPFC (**Extended Data Fig. 6h**). FISH demonstrated that most of the LH-projecting neurons express *Slc17A7* (gene encoding vesicular glutamate transporter 1) and are thus mainly excitatory (**Extended Data Fig. 6i** top**,j**). In contrast, *Cre*-expressing mPFC neurons hardly overlapped with cells expressing *Slc32a1* (gene encoding vesicular GABA transporter) (**Extended Data Fig. 6i** middle**,j**). The mPFC→LH population also expressed the gene *Npr3* (**Extended Data Fig. 6i** bottom**,j**), which is enriched in layer 5B and has been previously shown to be expressed in LH-projecting mPFC Pyr neurons^27,28^. Thus, these experiments identify the mPFC→LH neurons as a subpopulation of mainly excitatory, *Npr3*-expressing Pyr neurons.

**Figure 4:**
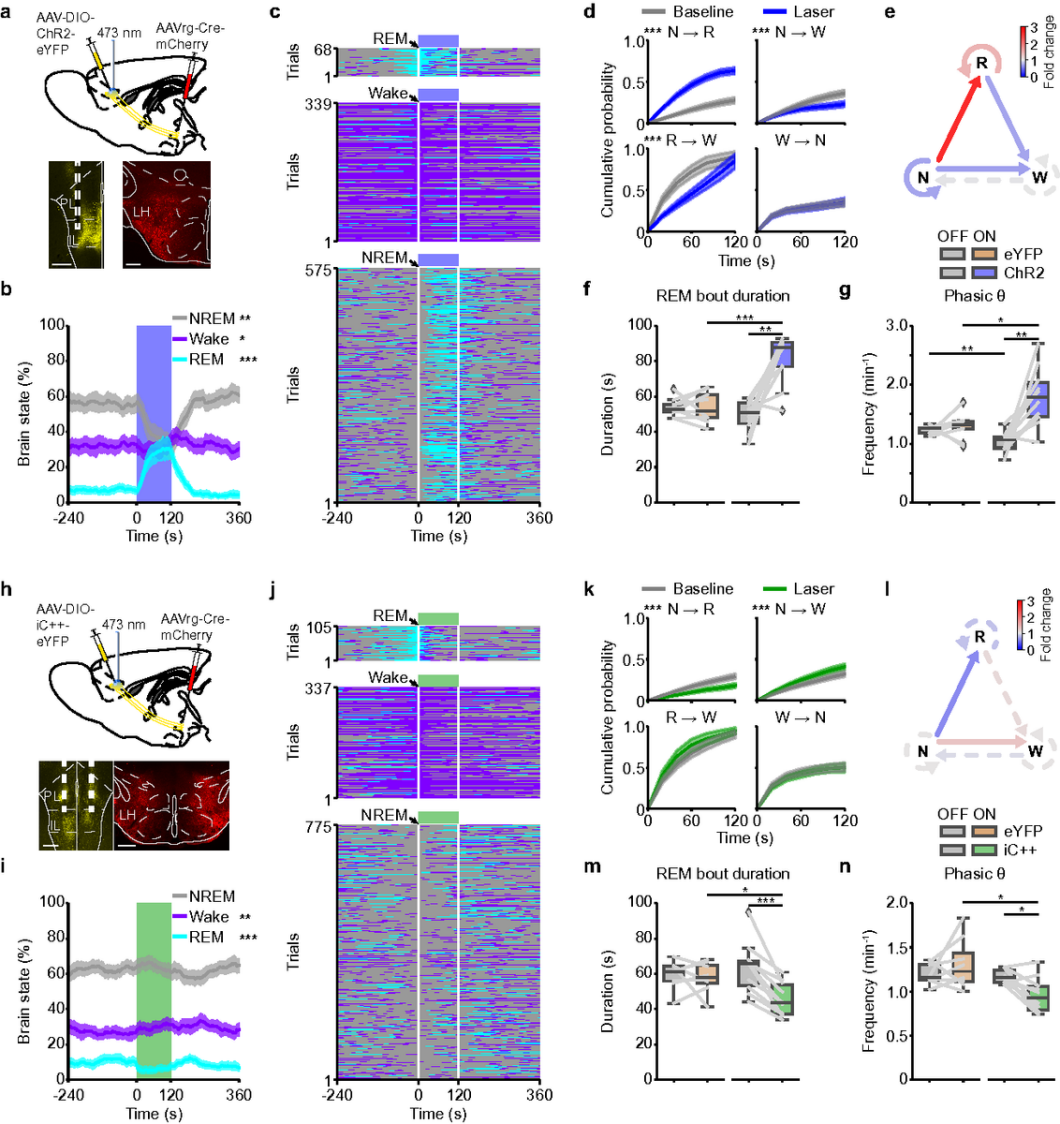
The effects on REM sleep and phasic θ events are mediated by LH-projecting mPFC neurons. (**a**) Schematic illustrating optogenetic activation of mPFC→LH neurons. Top, C57BL/6J mice were injected with AAVrg-Cre-mCherry into the lateral hypothalamus (LH) and AAV-DIO-ChR2-eYFP into the mPFC, followed by implantation of an optic fiber into the mPFC. Bottom, expression of ChR2-eYFP in the mPFC (left) and Cre-mCherry in the LH (right). Scale bars, 500 μm.. (**b**) Percentages of brain states before, during, and after open-loop stimulation. Blue bar, laser stimulation interval. Two-way rm ANOVA (interaction, P = 0.0002); t-tests with Holm-Bonferroni correction; baseline vs laser: REM, P = 0.0006; wake, P = 0.0478; NREM, P = 0.0013. n = 10 mice. Lines, averages across mice; shadings, 95% CIs.. (**c**) Laser stimulation trials from all n = 10 mice, sorted depending on the brain state at laser onset (t = 0 s, arrows).. (**d**) Cumulative transition probabilities during baseline and laser interval for open-loop activation of mPFC→LH neurons. Bootstrap; N→R, P = 0.0001; R→W, P = 0.0001; N→W, P = 0.0001; W→N, P = 0.8128; n = 10 mice. Shadings, 95% CIs.. (**e**) Graph summarizing laser-induced changes in the cumulative transitions probabilities.. (**f**) Left, effect of closed-loop activation of mPFC→LH neurons on REM sleep duration. Mixed ANOVA (laser, P = 0.0002; interaction, P = 0.0004); t-tests with Holm-Bonferroni correction; OFF vs ON: eYFP, P = 0.9388; ChR2, P = 0.0014; eYFP vs ChR2: OFF, P = 0.2100; ON, P = 0.0003. ChR2, n = 10; eYFP, n = 9 mice. Box plots; lines, individual mice.. (**g**) Frequency of phasic θ events during REM sleep episodes with (ON) and without closed-loop activation (OFF). Mixed ANOVA (laser, P = 0.0007; interaction, P = 0.0034); t-tests with Holm-Bonferroni correction; OFF vs ON: eYFP, P = 0.5128; ChR2, P = 0.0043; eYFP vs ChR2: OFF, P = 0.0066; ON, P = 0.0138. ChR2, n = 10; eYFP, n = 9 mice. Box plots; lines, individual mice.. (**h**) Schematic illustrating optogenetic inhibition of mPFC→LH neurons. Top, C57BL/6J mice were injected with AAVrg-Cre-mCherry into the LH and with AAV-DIO-iC++-eYFP into the mPFC, followed by bilateral implantation of optic fibers into the mPFC. Bottom, expression of iC++-eYFP in the mPFC (left) and Cre-mCherry in the LH (right). Scale bars, 500 μm.. (**i**) Percentages of brain states before, during, and after open-loop inhibition. Green bar, laser stimulation interval (473 nm, 120 s step pulse). Two-way rm ANOVA (interaction, P = 0.0022); paired t-tests with Holm-Bonferroni correction; baseline vs laser: REM, P = 0.0000; wake, P = 0.0088; NREM, P = 0.1668. n = 10 mice. Lines, averages across mice; shadings, 95% CIs.. (**j**) Laser stimulation trials from all n = 10 mice.. (**k**) Cumulative transition probabilities during baseline and laser interval for open-loop inhibition of mPFC→LH neurons. Bootstrap; N→R, P = 0.0001; R→W, P = 0.0622; N→W, P = 0.0002; W→N, P = 0.3630; n = 10 mice. Shadings, 95% CIs.. (**l**) Graph visualizing changes in the cumulative transitions probabilities induced by mPFC→LH inactivation.. (**m**) Left, effect of closed-loop inhibition of mPFC→LH neurons on REM sleep duration. Mixed ANOVA (laser, P = 0.0004; interaction, P = 0.0033); t-tests with Holm-Bonferroni correction; OFF vs ON: eYFP, P = 0.7220; iC++, P = 0.0005; eYFP vs. iC++: OFF, P = 0.4493; ON, P = 0.0212. iC++, n = 10; eYFP, n = 8 mice. Box plots; lines, individual mice.. (**n**) Frequency of phasic θ events during REM sleep episodes with and without closed-loop inhibition. Mixed ANOVA (laser, P = 0.2558; interaction, P = 0.0108); t-tests with Holm-Bonferroni correction; OFF vs ON: eYFP, P = 0.2931; iC++, P = 0.0143; eYFP vs iC++: OFF, P = 0.5441; ON, P = 0.0244; iC++, n = 10; eYFP, n = 8 mice. Box plot; lines, individual mice.. Brain atlas images in (**a,h**) adapted with permission from ref.^61^. See **Supplementary Table 1** for detailed statistical information. *P<0.05, **P<0.01, ***P<0.001.

Combined injection of AAVrg-Cre-mCherry into the LH and of Cre-dependent AAVs encoding ChR2-eYFP into the mPFC allowed for expression of ChR2-eYFP in the mPFC→LH neurons (Fig. 4a, **Extended Data Fig. 8a**). Optogenetic activation strongly increased the percentage of REM sleep during laser stimulation at the expense of NREM sleep (Fig. 4b,c, **Extended Data Fig. 8e**), at magnitudes comparable with those found for mPFC Pyr neuron stimulation (Fig. 1d,e). Consistent with these brain state changes, stimulation of mPFC→LH neurons caused an increase in the θ and γ power and lowered the δ power during the laser interval (**Extended Data Fig. 8b**). In eYFP mice, laser stimulation had no effect (**Extended Data Fig. 8c,d**), and the laser-induced changes in the percentages of REM and NREM sleep consequently differed between ChR2 and eYFP mice (**Extended Data Fig. 8e**).

Optogenetic stimulation of the LH-projecting neurons strongly enhanced the cumulative probability of NREM→REM transitions throughout the laser interval, while it reduced NREM→Wake transitions to a lesser degree, weakening the maintenance of NREM episodes (Fig. 4d,e, **Extended Data Fig. 8f,g**). As observed for activation of mPFC Pyr neurons (Fig. 1g,h), stimulation of mPFC→LH neurons reduced the likelihood of REM→Wake transitions and consequently maintained REM sleep (Fig. 4d,e, **Extended Data Fig. 8f,g**). In contrast to mPFC Pyr neuron stimulation, Wake →NREM transitions and the probability to remain in wake were not affected by activation of mPFC→LH neurons (Fig. 4d,e, **Extended Data Fig. 8f,g**), suggesting that mPFC→LH neuron activation specifically promotes REM sleep.

As found for mPFC Pyr neuron activation, closed-loop stimulation of mPFC→LH neurons prolonged REM sleep episodes, compared with the duration of laser-off episodes in the same animals and compared with laser-on episodes in eYFP controls (Fig. 4f). In ChR2-expressing animals, the laser-on duration also differed from the duration of REM sleep in baseline recordings without laser (**Extended Data Fig. 8h**). Compared with eYFP controls, activation of mPFC→LH neurons during REM sleep reduced the EEG δ power (**Extended Data Fig. 8i**) and strongly increased the frequency of phasic θ events (Fig. 4g). These results demonstrate that activation of the mPFC→LH subpopulation alone was sufficient to induce and maintain REM sleep as well as to facilitate phasic θ events. To further probe the robustness of these findings, we tested whether the activity of these neurons also promotes REM sleep under different stimulation conditions. First, we verified that optogenetic activation with lower frequency (2.5 Hz instead of 5 Hz) also reliably induces and maintains REM sleep episodes and enhances phasic θ events (**Supplementary Fig. 6a–c**). Activation of mPFC→LH neurons for 60 s (instead of 120 s) was sufficient to strongly initiate REM sleep (**Supplementary Fig. 6d**). Finally, despite lower levels of sleep during the dark phase, excitation of mPFC→LH neurons for 60 s (instead of 120 s) was sufficient to strongly initiate REM sleep (**Supplementary Fig. 6d**). Finally, despite lower levels of sleep during the dark phase, excitation of mPFC→LH during the active phase also enhanced REM sleep and increased the frequency of phasic θ events (**Supplementary Fig. 6e–g**).

In contrast to ChR2-mediated activation, optogenetic inhibition of mPFC→LH neurons using iC++ (Fig. 4h, **Extended Data Fig. 9a**) lowered the percentage of REM sleep during the laser interval, while increasing the percentage of wakefulness (Fig. 4i,j). There was no significant effect in eYFP mice (**Extended Data Fig. 9c–d**), and the laser-induced changes in the brain state differed between iC++ and eYFP mice (**Extended Data Fig. 9e**). The laser-trial averaged EEG spectrogram exhibited a small reduction in the θ and γ power and increase in the δ power (**Extended Data Fig. 9b**). Inhibition suppressed NREM→REM transitions and instead increased transitions to wakefulness, underlying the reduction in REM sleep (Fig. 4k,l, **Extended Data Fig. 9g**). Compared with laser-off episodes in iC++ animals and laser-on episodes in eYFP controls, closed-loop inhibition shortened REM sleep bouts (Fig. 4m) and reduced the frequency of phasic θ events (Fig. 4n). The decrease in the REM sleep duration was also significant in comparison with the REM sleep duration in baseline recordings from the same animals without laser stimulation (**Extended Data Fig. 9h**). In contrast to mPFC Pyr neuron inhibition (**Extended Data Fig. 3f**), inactivating mPFC→LH neurons did not attenuate the θ power during REM sleep (**Extended Data Fig. 9i**), indicating that the effects on the θ power and phasic θ events are likely mediated by different subpopulations in the mPFC.

To examine how sustained inhibition of mPFC→LH neurons affects REM sleep, we performed chemogenetic inhibition experiments by expressing hM4D(Gi) in these neurons by injecting AAVrg-Cre into the LH and AAV-DIO-hM4D(Gi) into the mPFC (**Extended Data Fig. 9j,k**). As high doses of the agonist clozapine-N-oxide (CNO) can affect sleep-wake states^29^, we directly compared the effects of chemogenetic inhibition in hm4D(Gi) mice with those in CNO-injected control mice expressing mCherry. CNO injection reduced the amount of REM sleep in hM4D(Gi) mice (**Extended Data Fig. 9l**), due to shortened REM sleep episodes (**Extended Data Fig. 9m**). The percentage of NREM sleep was elevated in hM4D(Gi) mice, and consequently, the ratio of REM sleep to total sleep was reduced (**Extended Data Fig. 9l**). Consistent with the results for optogenetic inhibition of mPFC→LH neurons, the frequency of phasic θ events was reduced (**Extended Data Fig. 9n**). Altogether, the results from opto- and chemogenetic manipulation support an important role of the mPFC→LH neuron activity in the regulation of REM sleep and phasic θ events.

### Activity of LH-projecting mPFC neurons during sleep

**Figure 5:**
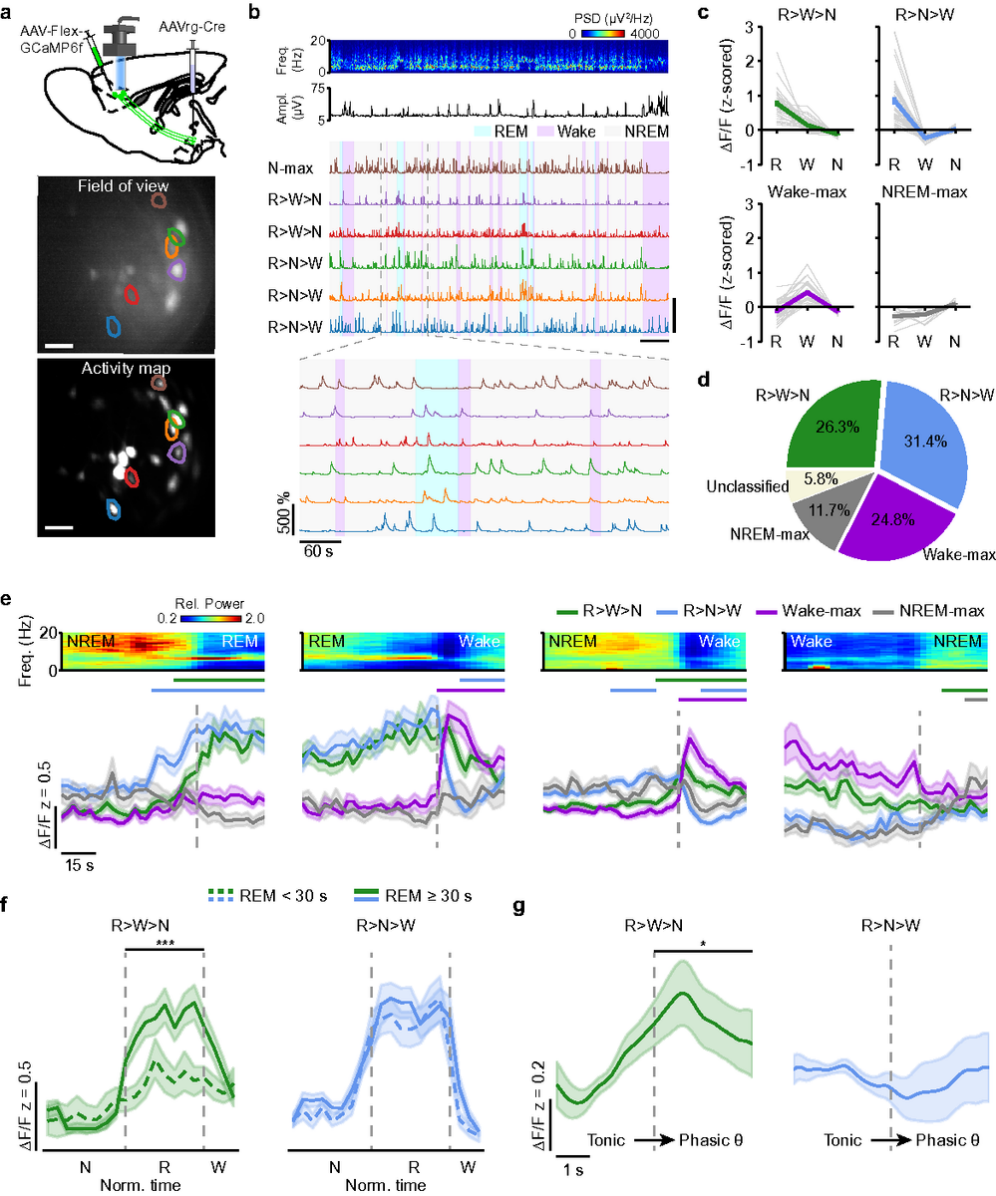
Activity of LH-projecting mPFC neurons during sleep. (**a**) Top, schematic of calcium imaging of mPFC→LH neurons using microendoscope. Bottom, field of view and pixel-wise activity map of an example imaging session. Colored polygons, example regions-of-interest (ROIs). Scale bars, 50 μm.. (**b**) EEG power spectrogram, EMG amplitude, brain states, and ΔF/F traces for the cells (ROIs) outlined in (**a**). The dashed lines indicate a region in which the ΔF/F signals are shown on an expanded time scale. The subclasses of the shown cells are indicated on the right. Scale bars, 300 s and 500%.. (**c**) Average calcium activity (ΔF/F, z-scored) of different cell subclasses during each brain state (R>W>N, n = 36; R>N>W, n = 43; Wake-max, n = 34; NREM-max, n = 16 cells; n = 8 mice). Bold lines, mean across cells ± s.e.m.; gray lines, individual cells.. (**d**) Proportion of different cell subclasses in the population of mPFC→LH neurons.. (**e**) Average EEG spectrogram (normalized by the mean power in each frequency band) and mean calcium activity (ΔF/F, z-scored) of the different cell subclasses at brain state transitions. Horizontal lines indicate for each subclass time points for which the ΔF/F activity significantly differed from baseline (**Supplementary Table 3;** Methods). R>W>N, n = 36; R>N>W, n = 43; Wake-max, n = 34; NREM-max, n = 16 cells. Lines, averages across cell subclasses; shadings, ± s.e.m.. (**f**) Activity of R>W>N and R>N>W cells during short (< 30 s) and long (≥ 30 s) REM sleep episodes. The duration of REM (R), wake (W), and NREM (N) episodes were normalized in time. Two-way rm ANOVA with REM duration (short or long; R>W>N: P = 0.0057, R>N>W: P = 0.4282) and brain state as within factors (interaction; R>W>N: P = 0.0014, R>N>W: 0.3656); t-tests with Holm-Bonferroni correction for REM; R>W>N: P = 0.0002. R>W>N, n = 36; R>N>W, n = 43 cells. Lines, mean across cells; shadings, ± s.e.m.. (**g**) Activity of different cell subclasses at transitions from tonic REM to phasic θ events (onset at t = 0 s). Paired t-test comparing mean activity during preceding tonic REM and phasic θ event; R>W>N, P = 0.0232; R>N>W, P = 0.7471. R>W>N, n = 36; R>N>W, n = 43 cells. Lines, mean across cells; shadings, ±s.e.m.. See **Supplementary Table 1** for detailed statistical information. *P<0.05, ***P<0.001.

To monitor the activity of mPFC→LH neurons during spontaneous sleep, we performed cellular-resolution calcium imaging (Fig. 5a,b). To express the calcium indicator GCaMP6f in the mPFC→LH neurons, we injected AAVrg-Cre into the LH and AAV-DIO-GCaMP6f into the mPFC (Fig. 5a, **Extended Data Fig. 10a**). Imaging was carried out in freely moving mice through a gradient refractive index (GRIN) lens coupled to a miniaturized fluorescence microscope with concurrent EEG and EMG recordings (Methods). The calcium activity of most of the imaged neurons significantly varied across brain states (Fig. 5b, **Supplementary Fig. 7**; 136/137 cells; one-way ANOVA, P < 0.05; **Supplementary Video 3**). Based on their state-dependent activity profile, we divided the recorded neurons into four different subclasses (Fig. 5c, **Supplementary Table 2;** Methods): Two subclasses comprised cells that were most active during NREM sleep (NREM-max) or wakefulness (Wake-max). Following previous studies^30,31^, we subdivided neurons that were most active during REM sleep (REM-max), which comprised the majority of the imaged cells (57.7%), into two further subclasses depending on whether they were more active during NREM sleep than wake (R>N>W) or vice versa (R>W>N). Among all subclasses, the R>N>W neurons formed with 31.4% of cells the largest subpopulation, followed by R>W>N neurons with 26.3% of cells (Fig. 5c,d). Wake-max neurons comprised 24.8% of the neurons, and NREM-max neurons were the smallest subclass with 11.7% of cells and showed the weakest modulation across brain states (Fig. 5c,d).

Analyzing the calcium activity of mPFC→LH neurons at brain state transitions, we found that both R>W>N and R>N>W neurons showed an increase in their activity before NREM→REM transitions (Fig. 5e; Methods). The activity of R>N>W neurons started significantly rising earlier than the activity of R>W>N neurons before the transition to REM sleep (Fig. 5e; **Supplementary Table 3a**) and rapidly decayed at the end of REM sleep, while that of R>W>N neurons displayed a more gradual decline (Fig 5e; **Supplementary Table 3b**). During NREM→Wake transitions, the activity of R>N>W neurons also increased throughout NREM sleep, but immediately decayed as the animal transitioned to wake (Fig. 5e; **Supplementary Table 3c**). This is in contrast to the R>W>N neurons, whose activity increased at the transition to wakefulness (Fig. 5e). During Wake→NREM transitions, the decaying activity pattern of R>W>N neurons resembled that of Wake-max neurons, while the temporal profile of R>N>W neurons was similar to that of NREM-max neurons, further differentiating the two subpopulations of REM-max neurons (Fig. 5e; **Supplementary Table 3d**). The calcium signal of the Wake-max neurons significantly rose only after the transition from NREM or REM sleep to wakefulness (Fig. 5e; **Supplementary Table 3b,c**), indicating that the endogenous activity of this subpopulation does not promote transitions to wakefulness, consistent with our observation that optogenetic activation of mPFC→LH neurons did not induce NREM→Wake or REM→Wake transitions (see Fig. 4d,e).

A previous study showed that the EEG θ and σ power during NREM sleep are correlated with the propensity to enter REM sleep^32^. Consistent with this, optogenetically increasing the number of sleep spindles, which strongly contribute to the σ power^33^, elevated the chance of NREM→REM transitions^34^. We therefore calculated for each subclass the cross-correlation between the θ or σ power and the calcium activity of its neurons during NREM sleep (Methods). Only the activity of R>N>W neurons was positively correlated with the θ and σ power, while the R>W>N and Wake-max neurons showed a negative correlation with these frequency bands (**Extended Data Fig. 10b**). Thus, the increase of the R>N>W neuron activity prior to the REM onset (Fig. 5e) and the positive correlation of their activity with both frequency bands suggests a role of this subclass in promoting transitions to REM sleep.

Because our optogenetic experiments demonstrated a role of the mPFC→LH neuron activity in maintaining REM sleep (Fig. 4f), we examined whether the activity of REM-max neurons during REM sleep was correlated with the REM episode duration. We found that the activity of R>W>N neurons, but not of R>N>W neurons, was positively correlated with the REM duration (**Extended Data Fig. 10c**), and their activity was consequently higher during long REM episodes than during short ones (Fig. 5f). These findings imply a potential role of the R>W>N neuron activity in maintaining REM sleep.

Since optogenetic activation of mPFC→LH neurons promoted phasic REM sleep, we studied how the calcium activity of mPFC→LH neurons changes during phasic θ events. Among all subclasses, only the R>W>N neurons showed a significant increase in the mean activity during phasic θ events compared with the preceding baseline activity (Fig. 5g, **Extended Data Fig. 10d;** Methods), indicating that specifically this subpopulation functionally contributes to the regulation of θ events. Thus, differences in their activity during phasic θ events and state transitions as well as differences in the correlation with the REM duration and with the θ and σ power suggest that R>N>W and R>W>N neurons constitute two distinct subgroups of REM-max neurons with different functional roles in REM sleep regulation.

### Presynaptic inputs to mPFC→LH neurons

**Figure 6:**
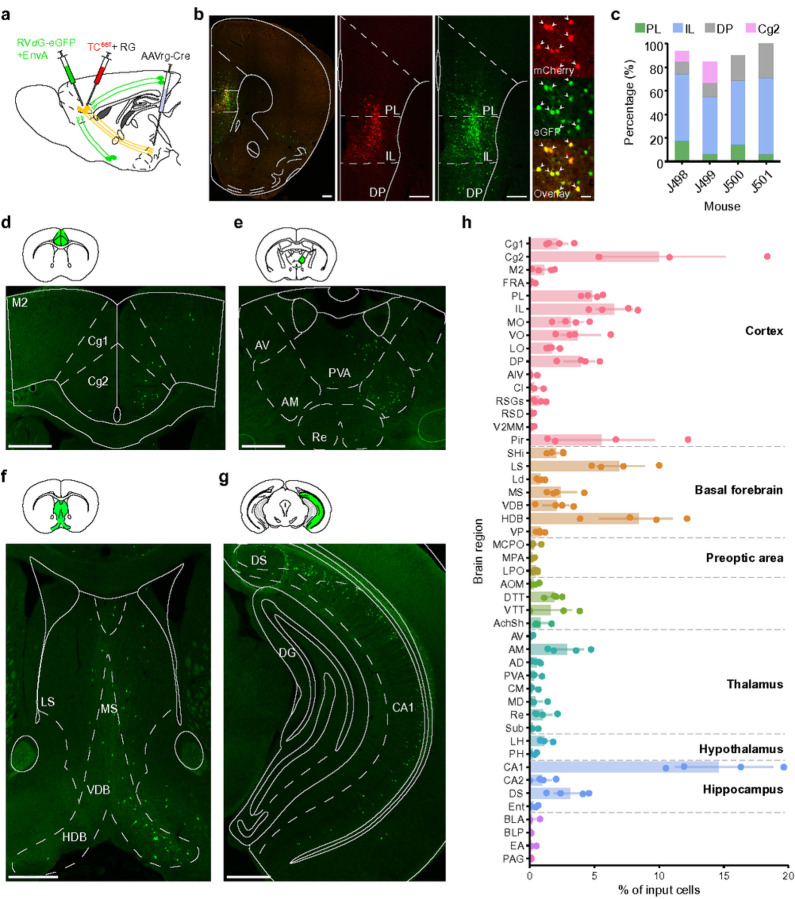
Presynaptic inputs of LH-projecting mPFC neurons. (**a**) Schematic illustration of rabies-mediated tracing of monosynaptic inputs to mPFC→LH neurons. Cre-recombinase was expressed in the mPFC→LH neurons by injecting AAVrg-Cre into the LH. For transsynaptic tracing, AAVs expressing a mutant EnvA receptor fused with mCherry (TC66T) and rabies glycoprotein (RG) were injected into the mPFC, followed by injection of the RG-deleted rabies virus expressing eGFP (RV*d*G-eGFP).. (**b**) Left, fluorescence image showing the location of starter cells in mPFC. Scale bar, 500 μm.. Middle, cells expressing TC66T (red) and eGFP (green) in mPFC. Scale bars, 500 μm. Right, enlarged view of starter cells expressing both TC66T and eGFP (white arrowheads). Scale bars, 25 μm. DP, dorsal peduncular cortex. Brain atlas images adapted with permission from ref.^61^.. (**c**) Distribution of starter cells within different mPFC subregions in all animals (n = 4 mice). Cg2, cingulate cortex area 2.. (**d–g**) RV-eGFP labeled cells in cingulate cortex (d), thalamic regions (e), septum and basal forebrain (f), and ventral hippocampus (g). The location of each fluorescence image within the mouse brain is indicated by the schematic brain sections on top. M2, secondary motor cortex; Cg1, cingulate cortex area 1; AV, anteroventral thalamic nucleus; AM, anteromedial thalamic nucleus; Re, reuniens thalamic nucleus; PVA, paraventricular thalamic nucleus, anterior part. LS, lateral septal nucleus; MS, medial septal nucleus; VDB, nucleus of the vertical limb of the diagonal band; HDB, nucleus of the horizontal limb of the diagonal band. DS, dorsal subiculum; DG, dentate gyrus. Scale bars, 500 μm.. (**h**) Proportion of RV-eGFP labeled inputs of mPFC→LH neurons across brain regions (see **Supplementary Table 4** for definition of brain region abbreviations). Bars, averages across mice; error bars, 95% CI; dots, individual mice; n = 4 mice.

The mPFC is known to integrate inputs from multiple cortical and subcortical areas^35^. To identify presynaptic neurons that specifically innervate the mPFC→LH subpopulation, we mapped its monosynaptic inputs using mono-synaptically restricted rabies tracing(Fig. 6a)^36^. Cre-recombinase was expressed in the mPFC→LH neurons by injecting AAVrg-Cre into the LH and Cre-inducible AAVs encoding the TVA receptor fused with mCherry (TC66T) and rabies glycoprotein (RG) were injected into the mPFC. A modified rabies-virus expressing eGFP (RV*d*G-eGFP+EnvA) was injected three weeks later into the mPFC. The majority of starter cells expressing both TC66T and eGFP were located in the mPFC (Fig. 6b,c). Most subcortical input neurons were found in the basal forebrain, known to be strongly activated during REM sleep, and the septum, involved in the regulation of θ oscillations during REM sleep (Fig. 6f,h)^3^. Presynaptic neurons in the thalamus were largely located in the anteromedial thalamic nucleus (AM) (Fig. 6e,h). Among cortical areas, the mPFC→LH neurons received most inputs from the cingulate cortex (Fig. 6d,h), as well as inputs from local neurons within the mPFC. Interestingly, most presynaptic neurons were localized in the ventral CA1 area (Fig. 6g,h). Thus, in addition to the mPFC, further presynaptic, REM sleep-active neurons in cortex, such as the cingulate cortex or the hippocampus^4,11,30^, may modulate REM sleep and phasic events through their projections to the mPFC and thus constitute a distributed cortical network regulating REM sleep.

## DISCUSSION

Our study demonstrates that the mPFC possesses the capability to exert top-down control of REM sleep via its projections to the LH and extends the traditionally held view that REM sleep is primarily regulated by subcortical circuits. Thus, while the circuits necessary for the generation of REM sleep and its defining features are localized in the brainstem, a distributed network spanning brainstem, hypothalamus, and cortex regulates the induction and maintenance of REM sleep as well as its composition of tonic and phasic substates.

There is growing evidence that the neural activity during REM sleep strongly differs between cortical areas, layers, and cell types. Recent mesoscale calcium imaging studies of the dorsal cortical surface revealed an activation of the occipital cortex centered around the retrosplenial cortex (RSC), while the activity in frontal areas was reduced^24,25^. In the somatosensory cortex and dorsal PFC, Pyr neurons show reduced activity during REM sleep^37^, likely as a result of somatic inhibition by REM sleep-active parvalbumin interneurons^38^. In contrast to these areas, in the motor cortex, the majority of Pyr neurons are most active during REM sleep^31^, comparable with the large fraction of REM-max neurons found in the retrosplenial and cingulate cortex^30^. While in these cortical areas the subclass of R>W>N neurons comprised most of the recorded neurons^30,31^, the R>N>W neurons formed the largest subgroup within the mPFC→LH population. Among the different subgroups of mPFC→LH neurons, the activity of the R>N>W neurons started increasing earliest before the onset of REM sleep, indicating that this subpopulation is functionally most relevant for the induction of REM sleep. The R>N>W neurons were the only subpopulation of mPFC→LH neurons showing positive correlation with the σ power during NREM sleep, which is strongly modulated by the frequency of sleep spindles^33^. Since optogenetically promoting sleep spindles facilitates transitions to REM sleep^34^, we speculate that the R>N>W subpopulation may relay spindle-related activity from the thalamocortical system via the LH to the brainstem, thus providing a mechanistic link between an increased spindle frequency and a higher chance to enter REM sleep^32,34^.

Previous studies reported a strong activation of the thalamocortical system during phasic REM sleep in humans^39^ and an increased firing rate of CA1 neurons in rats^11^. In the RSC, a subgroup of layer 2/3 neurons has been recently shown to be specifically activated during the initial phase of REM sleep, where EMs are rare, and inactivation of these neurons delayed the onset of EMs^24^. Together with our finding that mPFC Pyr neurons directly regulate the density of both EMs and phasic θ events, these results highlight a surprising role of the cortex in coordinating the timing and density of phasic events. Among all subclasses of mPFC→LH neurons, only the R>W>N neurons showed a significant increase in their activity during phasic θ events. Given the role of these events in facilitating inter-areal synchronization^8,11^, it would be interesting to test whether manipulation of the R>W>N neurons disrupts communication between mPFC and other areas during REM sleep and thereby impairs learning.

Among the various cell types in the LH involved in sleep-wake regulation^3^, one candidate population likely excited by the mPFC→LH neurons are the melanin concentrating hormone (MCH) neurons^40,41^, which are known to promote REM sleep upon opto- and chemogenetic activation^3^. In addition, MCH neurons can modulate hippocampal activity through direct projections to the hippocampus or indirectly via the lateral septum^42,43^,and interactions of the mPFC→LH subpopulation with MCH neurons may therefore contribute to its facilitatory effect on phasic θ events.However, in contrast to the reduction in REM sleep that we observed for silencing mPFC→LH neurons, optogenetic inhibition of MCH neurons does not decrease REM sleep or shorten its duration^44,45^, suggesting the involvement of further LH subpopulations mediating the effects of the mPFC on REM sleep. Particularly the posterior LH contains a large number of inhibitory REM sleep-activated neurons^46,47^, which are thought to promote REM sleep through their projections to REM sleep-suppressing neurons in the midbrain, including the ventrolateral periaqueductal gray (vlPAG), the adjacent deep mesencephalic reticular nucleus (DpMe), and dorsal raphe (DR)^46,48^. These midbrain areas also receive inhibitory inputs from REM sleep-promoting neurons in the ventral and dorsomedial medulla, and activation of the axonal projections from these medullary neurons to the midbrain has been shown to promote REM sleep^49,50^. A key question for future research is whether there exists a clear hierarchy in the REM sleep circuitry with specific first-order neurons initiating REM sleep, or whether multiple neural populations, distributed across various areas, form a brain-wide network, in which each node can trigger REM sleep independently. Of note, we cannot exclude that collaterals of the mPFC→LH neurons projecting to other areas besides the LH^17^ may also contribute to the observed effects on REM sleep and that other mPFC subpopulations with different postsynaptic targets also play a role in sleep-wake regulation. For instance, prefrontal projections to the dorsomedial hypothalamus have been reported to promote wakefulness^51^. A previous study showed that in orexin/hypocretin knock-out mice activation of mPFC neurons by palatable food increases the number of cataplectic attacks^41^. It would be interesting to study to what extent the involved neurons overlap with the REM sleep-promoting mPFC→LH neurons, and whether they innervate the same downstream targets or interact with other structures implicated in cataplexy such as the amygdala^52–54^.

Besides REM sleep- and wake-active neurons, the mPFC→LH subpopulation also comprises a smaller population of NREM-max neurons. It would be interesting to test whether other cortical areas also contain such NREM-active projection neurons, potentially in larger numbers, as synaptic silencing of a subset of layer 5 neurons throughout the whole cortex reduced NREM sleep^55^. Interestingly, the human vmPFC, which is strongly activated during REM sleep^2,5^, shows hyperactivity in depressed patients^13^. Thus, our finding that mPFC activation promotes REM sleep and phasic events may mechanistically explain the shortened latency to REM sleep, its increased duration, and the increased EM density characteristic of sleep in depression^12^. Consistent with the association between vmPFC hyperactivity and depression, bilateral lesions in the vmPFC are correlated with lower levels of depression^56^ and recovery from depression is associated with a reduction in vmPFC activity in human patients^57^. Similarly, pharmacological inhibition of the mPFC in rats reduced depressive-like behaviors^58^. In contrast, a previous study in rats found that lesions in the mPFC increase depressive-like behaviors and enhance REM sleep^59^. This discrepancy may result from differences in the exact location of the targeted areas. Second, as shown in our study, excitatory and inhibitory mPFC neurons have opposing effects on REM sleep and consequently, the extent to which either neuron type is lesioned or inhibited may induce opposing changes in sleep behavior as well as depressive symptoms. The simultaneous inactivation of both inhibitory and excitatory neurons may also explain why in a previous study non-cell type specific chemogenetic inhibition of the mPFC did not alter the amount of REM sleep^60^. For the future, understanding the distinct roles of different cortical areas, layers, and cell types in sleep control may help to infer from disturbances in REM sleep and its characteristic phasic activity changes in the underlying prefrontal circuits and to improve existing biomarkers for the early onset detection of mood disorders.

## METHODS

### Animals.

All animal care and experimental procedures were approved by the Institutional Animal Care and Use Committee (IACUC) at the University of Pennsylvania and conducted in accordance with the National Institutes of Health Office of Laboratory Animal Welfare Policy. All experiments were performed in adult male and female C57BL/6J mice (Jackson Laboratory; stock no. 000664) and Vgat-IRES-Cre mice (stock no. 028862) which were aged 8 – 10 weeks old at the point of surgery. Mice were randomly assigned to experimental and control groups. Food and water were available ad libitum under a 12:12 hour light:dark cycle with light on from 07:00 to 19:00. The colony room was maintained at an ambient temperature of 20 – 23°C and humidity of 40 – 60%. Animals for fiber photometry, opto- and chemogenetic experiments were group-housed except during sleep recordings. Mice for microendoscope imaging were single-housed after GRIN lens implantation.

### Surgical Procedures.

All surgeries were performed following the IACUC guidelines for rodent survival surgery. After subcutaneous injection of meloxicam (5 mg/kg), mice were anesthetized with 1 – 4% (vol/vol) isoflurane in oxygen and positioned in a stereotaxic frame (David Kopf Instruments) on a heating pad to maintain the body temperature throughout the procedure. Following asepsis, the skin was incised to gain access to the skull.

For EEG recordings, stainless steel wires (A–M systems) were attached to two screws fixed to the skull on top of the frontal and parietal cortex, or on top of the left and right parietal cortex. The reference electrode was connected to a screw placed on top of the left cerebellum. For EMG recordings, two stranded stainless steel wires (A–M systems) were inserted into the neck muscles. All electrodes were connected before the surgery to a mini-connector, which was secured to the skull using dental cement. For sleep recordings in head-fixed animals, a headplate was additionally fixed to the skull with dental cement.

Viruses were injected using Nanoject II (Drummond Scientific) via a glass micropipette into the mPFC (anteroposterior (AP) +1.90 mm; mediolateral (ML) ±0.35 mm; dorsoventral (DV) −2.40 mm) or LH (AP −1.4 mm; ML ±0.95 mm; DV −5.2 mm). To optogenetically modulate mPFC Pyr neurons in C57BL/6J mice, AAV2-CaMKIIα-hChR2(H134R)-EYFP-WPRE (0.4 μl, University of North Carolina (UNC) vector core, 4.0 × 10^12^ gc/ml) was injected unilaterally into the mPFC for activation, whereas AAV2-Ef1a-DIO-iC++-EYFP (0.5 μl each, UNC vector core, 5.9 × 10^12^ gc/ml) and AAV9-CaMKII0.4-Cre-SV40 (0.1 μl each, University of Pennsylvania vector core, 2.9 × 10^13^ gc/ml) were injected bilaterally into the mPFC for inhibition. For optogenetic manipulation of LH-projecting mPFC neurons, AAVrg-Ef1a-mCherry-IRES-Cre (0.35 μl, Addgene, 1.7 × 10^13^ gc/ml) was injected unilaterally or bilaterally into the LH, followed by unilateral injection of AAV2-Ef1a-DIO-hChR2(H134R)-EYFP (0.4 μl, UNC vector core, 4.0 × 10^12^ gc/ml) for optogenetic activation or bilateral injection of AAV2-Ef1a-DIO-iC++-EYFP (0.4 μl, 5.9 × 10^12^ gc/ml) for inhibition into the mPFC. For the optogenetic activation of mPFC inhibitory neurons, AAV2-Ef1a-DIO-hChR2(H134R)-EYFP (0.4 μl, UNC vector core, 4.0 × 10^12^ gc/ml) was injected unilaterally into the mPFC of Vgat-IRES-Cre mice. For control, AAV2-Ef1a-DIO-EYFP (0.4 μl, Addgene, 5.6 × 10^12^ gc/ml) was instead injected into the mPFC of C57BL/6J or Vgat-IRES-Cre mice. After virus injection, a mono fiber optic cannula (200 μm diameter, Doric lenses) for optogenetic activation or a dual fiber optic cannula (200 μm diameter, Doric lenses) for inhibition was implanted into the mPFC (DV −2.35 mm). For activation of axonal mPFC projections, a mono fiber optic cannula was inserted into the ipsilateral LH, mediodorsal thalamus (AP −1.0 mm; ML ±0.4 mm; DV −2.7 mm), laterodorsal tegmental area (AP −5.0 mm; ML ±0.45 mm; DV −3.2 mm), or periaqueductal gray (AP −4.2 mm; ML ±0.5 mm; DV −2.2 mm).

For calcium imaging of mPFC Pyr neurons using fiber photometry, AAV9-CaMKII0.4-Cre-SV40 (0.1 μl, 2.9 × 10^13^ gc/ml) and AAV1-Syn-Flex-GCaMP6s-WPRE-SV40 (0.4 μl, Addgene, 3.1 × 10^12^ gc/ml) were unilaterally injected into the mPFC and the optic fiber (400 μm diameter) was implanted on the top of the injection site (DV −2.40 mm). For calcium imaging of LH-projecting mPFC neurons, AAV1-Syn-Flex-GCaMP6f-WPRE-SV40 (0.4 μl, Addgene, 1.3 × 10^13^ gc/ml) was injected into the mPFC unilaterally and AAVrg-hSyn-Cre-WPRE-hGH (0.35 μl, Addgene, 1.5 × 10^13^ gc/ml) was injected into the ipsilateral LH to express GCaMP6f in mPFC→LH neurons. 2 to 4 weeks after virus injection, a GRIN lens (500 μm diameter, Inscopix) was implanted into the mPFC (DV −2.35 mm).

For chemogenetic inhibition of mPFC→LH neurons, mice were bilaterally injected with AAVrg-Ef1amCherry-IRES-Cre (0.35 μl, UNC vector core, 1.7 × 10^13^ gc/ml) into the LH and AAV8-hSyn-DIO-hM4D(Gi)-mCherry (0.3 μl, UNC vector core, 1.2 × 10^13^ gc/ml) into the mPFC. Control animals were instead injected with AAV8-hSyn-DIO-mCherry (0.3 μl each, UNC vector core, 2.2 × 10^13^ gc/ml) into the mPFC.

For anterograde tracing of projections from mPFC pyramidal neurons, AAV9-CaMKII0.4-Cre-SV40 (0.1 μl, 2.9 × 10^13^ gc/ml) and AAV2-CAG-Flex-toTomato (0.25 μl, UNC vector core, 4.8 × 10^12^ gc/ml) were unilaterally injected into the mPFC. For rabies virus-mediated retrograde tracing, AAVrg-hSyn-Cre-WPRE-hGH (0.35 μl, Addgene, 1.5 × 10^13^ gc/ml) was unilaterally injected into the LH, and a 1:1 ratio mixture of AAV-CAG-FLEx^loxP^-TC^66T^ (1.0 × 10^12^ gc/ml) and AAV-CAG-FLEx^loxP^-RABV-G (1.0 × 10^12^ gc/ml) was injected into the ipsilateral mPFC (0.25 μl). Three weeks later, RVdG-eGFP+EnvA virus (0.3 μl, 5 × 10^8^ cfu/ml) was injected into the same mPFC location. Mice with no virus expression, where virus expression was outside the target site, or where the optic fiber implant was misplaced were excluded from the data set.

### Histology, immunohistochemistry, and in situ hybridization.

Mice were deeply anesthetized and transcardially perfused with 0.1 M phosphate-buffered saline (PBS) followed by 4% paraformaldehyde. Removed brains were fixed in PFA overnight and then stored in 30% sucrose (w/v) in PBS solution. For histology, brains were sliced into 20 μm sections for *in situ* hybridization or 40 μm sections using a cryostat (Thermo Scientific HM525NX) and mounted onto glass slides.

For quantification of retrograde rabies tracing, we collected brain sections at 120 μm intervals and counted the starter cells (labeled with both mCherry and eGFP) and presynaptic neurons (labeled with eGFP only) within brain regions as defined by the Franklin and Paxinos mouse brain atlas^61^. For each animal, the number of starter or presynaptic cells in each region was divided by the total number of starter or presynaptic cells in the entire brain.

For immunohistochemistry targeting eYFP-expressing axons, mounted brain sections were permeabilized in 0.5% Triton-X solution in PBS (PBST) for 1 hour and incubated in the blocking solution, composed of 5% normal donkey serum in 0.3% PBST for 1 hour. Sections were incubated with a chicken anti-GFP primary antibody (1:1000, Aves Lab, GFP-1020), diluted in PBS for overnight at 4°C and then washed in PBS before incubation with a donkey anti-chicken secondary antibody tagged with green Alexa fluorophore (1:500, Jackson Immuno Research Laboratories, Inc., 703-545-155) for 2 hours. After washing with PBS, brain sections were stained with Hoechst solution (33342, Thermo Scientific) and coverslipped with Fluoromount-G (Southern Biotechnic).

Fluorescence in situ hybridization (FISH) was performed using an RNAscope assay according to the manufacturer’s instructions (RNAscope^®^ Fluorescent Multiplex Reagent Kit, Cat. # 320850, Advanced cell Diagnostics). Sections were hybridized with a *Cre* (#312281) and *Slc17a1* (#416631), *Slc32a1* (#319191), or *Npr3* (#502991) probe, or *eYFP* (#312131) and *Slc17a1* probe, and amplification steps were carried out followed by Hoechst staining. Fluorescence images were taken using a fluorescent microscope (microscope, Leica DM68; camera, Leica DFC7000GT).

### Polysomnographic recordings.

For sleep recordings, each mouse was first habituated for two days to its specific recording cage, placed within a sound-attenuating chamber. Recordings were performed during the light cycle and animals were single-housed in their recording cages throughout the duration of the recordings. EEG and EMG signals were recorded using an RHD2132 amplifier (Intan Technologies, sampling rate 1 kHz) connected to the RHD USB Interface Board (Intan Technologies), which was controlled by the RHD Recording Controller software (Intan Technologies, version 1.5.2), or a TDT RZ5P amplifier (Tucker-Davis Technologies, sampling rate 1.5 kHz), controlled using TDT’s Synapse software. During the recordings, the EEG and EMG electrodes were connected to a flexible recording cable. Videos were recorded using a camera (FLIR, Chameleon3, or ELB, Mini USB camera) mounted above the mouse cage.

Brain states were scored manually by visual inspection of the EEG and EMG signals, EEG spectrogram, and EMG power using a graphical user interface programmed in Python (https://zenodo.org/deposit/8035420). Spectrograms of the EEG and EMG signals were computed using sliding, half-overlapping 5 s windows, resulting in 2.5 s time resolution of the hypnogram. To estimate within each 5 s window the power spectral density (PSD), we performed Welch’s method with Hanning window using sliding, half-overlapping 2 s intervals. States with low-amplitude, fast EEG activity and increased EMG tone were scored as Wake. States with dominant low frequency activity in the delta range and low EMG tone were classified as NREM. States with dominant theta oscillations and low EMG tone were scored as REM. The annotator was not blinded to the identity of the animal, but to the timing of the laser.

### Optogenetic stimulation.

Optogenetic stimulation experiments were performed 3 to 6 weeks after virus injection. Recordings were performed during the light cycle (9 am to 5 pm) after a 60 min habituation period and lasted for 8 hours on average. For each animal, we performed three open- and closed-loop recordings on separate days. Additional baseline recordings without laser stimulation were performed in a subset of animals. For optogenetic stimulation, a flexible patch cable connected with the laser was attached to the implanted optic ferrule in addition to the cable for EEG/EMG recordings. For optogenetic stimulation, a blue laser (Laserglow) received TTL pulses generated by a Raspberry Pi (https://github.com/justin0bk/socketrecv), which was controlled by a custom-programmed user interface (https://github.com/justin0bk/sleepRecording_v9). For open-loop stimulation, we randomly presented 120 s (or 60 s) pulse trains every 13 – 17 min or (12 – 16 min). For closed-loop stimulation, REM sleep was detected online based on real-time spectral analysis of EEG and EMG signals. For the REM detection, we calculated from previous recordings of the same animals thresholds for the delta power and EMG amplitude as well as a hard and soft threshold for theta/delta ratio. The onset of a REM sleep episode was defined as the time point where the delta power and EMG amplitude were lower than their respective thresholds and where the theta/delta ratio surpassed the hard threshold. Laser stimulation lasted until the REM episode ended, i.e. when the theta/delta threshold dropped lower than the soft threshold or when the EMG amplitude was larger than its threshold. To compare REM sleep episodes with and without laser stimulation, the laser was turned on for randomly selected 50% of REM sleep episodes. Validation of the algorithm for closed-loop REM sleep detection is provided in **Supplementary Fig. 2.**

For optogenetic open-loop or closed-loop activation of mPFC Pyr neurons, axonal projections and LH-projecting mPFC neurons, 5 Hz (5 ms up, 195 ms down) or 2.5 Hz (5 ms up, 395 ms down) laser pulse trains were emitted (473 nm, 1.5 – 3.0 mW at fiber tip; Laserglow). For the activation of mPFC Vgat neurons 20 Hz pulse trains (5 ms up, 45 ms down) were applied for open and closed-loop stimulation. To inhibit mPFC pyramidal neurons and LH-projecting mPFC neurons, a step-pulse (3.0 – 4.0 mW) was applied for 120 s for open-loop stimulation or throughout REM sleep episodes in closed-loop experiments.

### Chemogenetic manipulation.

For chemogenetic inhibition of mPFC→LH neurons, clozapine N-oxide dihydrochloride (CNO; 5 mg/kg, Tocris Bioscience) was injected intraperitoneally (i.p.) into experimental mice expressing hM4D(Gi) in mPFC→LH neurons or control mice expressing only mCherry. Each recording session started right after injection at 9 am and lasted for 4 hours during the light phase. There was a minimum of one day without CNO injection between two consecutive CNO recordings. We recorded 3 sessions for each animal and calculated for each mouse the average across these sessions for the analyses in **Extended Data Fig. 9j–n.**

### Head-fixation for video-oculography.

To track the pupil, mice were head-fixed by attaching the implanted headplate to a frame. The animal’s head was kept at a 30° angle to imitate a natural head position^62^. An infrared (IR) camera (chameleon 3, FLIR; 30 Hz frame rate) was placed in front of the animal’s eye. Tracking the pupil during REM sleep was possible, because mice typically sleep with open eyes when head-fixed^62^. Starting one week after surgery, mice were habituated to sleep well under head-fixation (**Supplementary Fig. 4**), by head-fixing them for increasing time intervals over the course of 14 days (starting from 30 min during the first up to 4 hours during the last days). To test whether manipulation of mPFC activity affects the frequency of EMs, we performed closed-loop stimulation experiments using the same protocol as in freely moving animals. In addition, we performed baseline recordings without laser stimulation in the same animals. Recording sessions were performed during the light cycle and lasted for 4 hours. For each animal we recorded four sessions on separate days.

### Fiber photometry imaging.

Fiber photometry was performed 3 to 6 weeks after virus injection, in mice freely moving in their recording cages, to which they were habituated for 2 days. For each animal, we performed four recording sessions on separate days. Flexible cables were connected to the optic fiber and EEG/EMG electrodes. Recordings were performed after 60 min of habituation and lasted up to 3 hours during the light cycle. To detect calcium signals, a first LED emitted the excitation wavelength of 465 nm with 210 Hz frequency. As a control for bleaching and movement artifacts, a second LED with 405 nm wavelength, independent of the intracellular calcium signals, was illuminated with 330 Hz frequency. Both lights were passed through dichroic mirrors before entering a patch cable, and fluorescent signals from GCaMP6s emission were collected and passed to a photoreceiver via dichroic mirrors and a GFP emission filter. Photoreceiver signals were then relayed to a TDT RZ5P amplifier and demodulated by TDT’s Synapse software into two signals corresponding to the 465 and 405 nm wavelengths. Using custom-written python scripts, both signals were first low-pass filtered at 2 Hz using a 4th order digital Butterworth filter. Next, using linear regression, we fitted the 405 nm to the 465 nm signal. Finally, the linear fit was subtracted from the 465 nm signal and the difference was divided by the linear fit yielding the ΔF/F signal. To determine the brain state, EEG and EMG signals were recorded together with fluorescence signals using the RZ5P amplifier.

### Statistical analysis of fiber photometry data.

To test during which brain state the average calcium activity of mPFC Pyr was highest, we performed one-way repeated-measures (rm) ANOVA, followed by pairwise t-tests (REM vs Wake, REM vs NREM, Wake vs NREM) with Holm-Bonferroni correction to account for multiple testing. To determine when the calcium activity of mPFC Pyr neurons started significantly increasing or decreasing before a NREM to REM sleep transition, we first determined for each mouse the average ΔF/F signal ranging from 60 s before to 30 s after the NREM→REM transition. We only included trials for which the preceding NREM sleep episode lasted at least 60 s (including microarousals, i.e. wake episodes ≤ 10 s). Using one-way rm ANOVA (with 10 s time bins as within factor), we tested whether the DF/F activity was significantly modulated throughout the analyzed time interval. Finally, using pairwise t-tests with Holm-Bonferroni correction we determined the time bins for which the activity significantly differed from the baseline bin (activity from −60 to −50 s).

### Microendoscopy imaging.

For cellular-resolution calcium imaging of LH-projecting mPFC neurons, 1 – 2 weeks after GRIN lens implantation, the base plate was fixed on the animal’s head on top of the lens using dental cement to attach the Miniscope V3.2 (Labmaker, Germany). Imaging sessions took place during the light cycle in the home cage placed within a sound-attenuating chamfiber and lasted for 1.5 hours. For each animal, we performed one or two imaging sessions on separate days. The miniscope was connected via a flexible cable to the Miniscope PCB and an additional flexible cable was attached to the mini-connector on the animal’s head for EEG/EMG recordings using an RHD2132 amplifier. The mice were habituated to the recording system for at least 1 hour after attaching the Miniscope to the baseplate. The miniscope camera was controlled using the Miniscope V3.2 data acquisition software (https://github.com/daharoni/Miniscope_DAQ_Software) and calcium imaging movies were acquired with a frame rate of 20 Hz.

### Imaging analysis.

To correct for lateral motion in the recorded calcium imaging videos, we created a spatially high-pass filtered image stack by subtracting from each image a spatially low-pass filtered version of itself^63^. We then manually selected a high-contrast area within the mean projection of the high-pass filtered image stack as spatial reference. For each movie frame in the high-pass filtered stack, we performed a 2D cross-correlation to determine the shift in the x- and y-direction optimizing the overlap between the current movie frame and the reference. To select regions of interest (ROIs) for further analysis, we first computed an activity map, M_x,y_, highlighting pixels with strong variations in their intensity over time using

$${\text{M}}_{\text{X},\text{Y}}={\langle \langle ({\text{f}}_{\text{X},\text{Y}}(\text{t})\;-\;{\text{f}}_{\text{X},\text{Y}})\;/\;({\text{f}}_{\text{X},\text{Y}}\;+\;{\text{f}}_{\text{avg}})\rangle \rangle }_{\text{w}}{}^{3}\rangle \text{t},$$

where f_x,y_(t) denotes the fluorescence at pixel (x,y) of movie frame t, f_x,y_ is the average of f_x,y_(t) over time, and f_avg_ refers to the average fluorescence across the whole movie. The notation ⟨…⟩_t_ depicts the temporal average, and ⟨…⟩_w_ is the output of a spatial 2×2 box filter. Cell body-shaped regions of interest (ROIs) on the activity map with high intensity were selected manually by encircling them with polygons using a custom-programmed graphical user interface. For each ROI we extracted a raw fluorescence trace (F(t)) as the average across pixels within that ROI for each frame. To correct for contamination of the fluorescence signal by out-of-focus neuropil^63^, we calculated for each ROI the neighboring neuropil signal, F_np_(t), within a bordering 10 μm broad ring with ~5 μm distance from the perimeter of each ROI, excluding other ROIs, and subtracted F_np_(t), scaled by a correction factor, c, from the raw ROI signal, F(t): F_subt_(t) = F(t) − c F_np_(t). The correction factor c was estimated for each recording session by calculating the ratio between the mean pixel intensity of a manually selected blood vessel, F_blood_vessel_, and a nearby region lacking an ROI signal, F_near_blood_vessel_, each subtracted by the mean pixel intensity of an off-lens region, F_off-lens_, i.e. c = (F_blood_vessel_ − F_off-lens_) / (F_near_blood_vessel_ − F_off-lens_). The baseline of each neuropil-subtracted fluorescence signal B(t) was estimated by calculating the linear regression fit to the values of F_subt_(t) for periods of low fluorescence activity, defined as values within the 20th percentile of each recording session^64^. Using the baseline, we calculated the relative change in fluorescence as a function of time using ΔF/F(t) = (F_subt_(t) - B(t)) / B(t). Finally, we extracted for each ROI a denoised fluorescence trace using the OASIS algorithm with an AR(1) model^65^ (**Supplementary Fig. 7d**). To identify the same ROIs within two imaging sessions, we first determined for each recording session a map with all ROIs and then rotated and translated these maps with respect to each other such that the overlap between the ROIs was maximized. ROIs from two imaging sessions with on overlap of at least 50% were interpreted as the same neuron. Calcium imaging recordings with weak fluorescence signals or no clearly detectable cell-body shaped ROIs were excluded from the data set.

### Definition of different mPFC→LH neuron subclasses.

For each ROI (cell), we tested whether its activity was significantly modulated by the brain state using one-way ANOVA (with ΔF/F values as dependent variable and brain state as factor). Using the Tukey’s HSD post-hoc test, we then determined for each ROI that was significantly modulated by brain state (P < 0.05), during which state the ΔF/F activity was highest. REM-max neurons were subdivided into two further subclasses, depending on whether they were more active during NREM sleep than wake (R>N>W) or vice versa (R>W>N).

### Calcium activity of mPFC→LH neurons during phasic θ events.

To test whether the calcium activity of cells within different subclasses was significantly modulated during phasic θ events, we calculated for each cell the mean activity during phasic θ events and the preceding baseline intervals of equal duration. For each subclass of cells, we then determined, using a paired t-test, whether their activity was significantly changed during phasic θ events.

### Analysis of mPFC→LH calcium activity at brain state transitions.

To calculate the calcium activity of the cells within each subclass during brain state transitions from state X to Y, we first aligned the ΔF/F signals of the cells for all X→Y transitions relative to the time point of the transition (t = 0 s). Next, we ensured that for each NREM→Y or Wake→Y transition the preceding NREM or wake episode lasted for at least 60 s. NREM episodes were allowed to be interrupted by MAs. In the case of REM→Wake transitions, the preceding REM sleep episode was at least 10 s long. Using one-way rm ANOVA, we tested whether the activity (downsampled to 10 s bins) within each subclass was significantly modulated throughout the transition (from −60 to 30 s). Finally, using pairwise t-tests with Holm-Bonferroni correction, we determined the time bins for which the activity significantly differed from the baseline bin (activity for bin −60 to −50s). To compare the activity of cells across multiple NREM→REM→Wake transition sequences (Fig. 5f), we normalized the duration of each NREM, REM, and wake episode and the corresponding ΔF/F signals by dividing them into fixed numbers of bins.

### Phasic θ event detection.

To detect phasic θ events in the EEG during REM sleep, we implemented a previously described algorithm^8,9,23^. For each REM sleep episode, the EEG was bandpass filtered in the range 5 – 12 Hz. To detect the troughs in the θ oscillations, we computed the instantaneous phase of the signal using the Hilbert transform and identified troughs as minima < −3 rad. Next, we calculated the time intervals between the troughs and filtered the sequence of intervals using an 11-element box filter. Following the definition in previous studies, a candidate epoch for a phasic θ event was a sequence of troughs, for which the smoothed sequence of inter-trough intervals was at least 900 ms long and continuously smaller than the 10th percentile of all smoothed inter-trough intervals. A candidate epoch was identified as a phasic θ event, if its minimum smoothed interval was smaller than the 5th percentile of the smoothed inter-trough intervals, and if the mean θ amplitude during the epoch was larger than the mean amplitude during entire REM sleep. The 10th, 5th percentile and the mean REM amplitude were calculated using all REM sleep episodes that did not overlap with laser stimulation.

### Heart rate detection.

The heart rate was calculated by detecting R-waves in the nuchal EMG (bandpass filtered between 10 – 100 Hz)^66^. First, we identified all REM sleep episodes for which R-waves could be well isolated (**Supplementary Fig. 3**). More precisely, we determined for each REM sleep episode whether there exists a threshold that well separates R-waves from background noise, by counting for increasing threshold values the number of negative peaks surpassing each threshold. Intuitively, for a very small threshold value no peak in the signal exceeds the threshold. But as the threshold rises, the number of detected peaks continuously increases. If the height of R-waves clearly differs from the noise, the number of detected peaks will reach a plateau (for threshold values between the R-wave and noise peaks), before sharply increasing, as the threshold starts including noise peaks. In case the R-waves are not well separated from the noise, the curve will rise without any clear plateau. As a criterion to determine whether R-waves can be well isolated, we therefore required that the threshold curve contains an inflection point in the range (−60 to 0 μV). If an inflection point existed, we included the corresponding REM sleep episodes in the dataset for the heart rate quantification and used it as threshold for the R-wave detection; otherwise the REM sleep episode was excluded. Mice with non-detectable R-waves in the EMG were excluded from the heart rate analysis.

### Pupil tracking.

To detect the pupil position in the recorded videos, we implemented an algorithm using opencv (https://pypi.org/project/opencv-python/). We inverted and smoothed each movie frame, and then applied binary thresholding to transform each frame (cropped to the area around the eye) to a 2D array of 1s and 0s. We then identified the pupil as the largest contour of 1s in the image, the center of which was defined as the pupil position. Using a graphical user interface we could manually verify and, if necessary, correct the pupil annotation. Next, we determined the pupil speed from its change in the x and y-direction between two successive movie frames (**Supplementary Fig. 5**). Using the speed, we then calculated the pupil acceleration for each time point. Rapid EMs were defined as positive peaks larger than two standard deviations of the acceleration (calculated across all REM sleep periods within a recording). Time points where the pupil was not visible due to blinking were excluded from the analysis.

### Calculation and statistical analysis of transition probabilities.

For the analysis of cumulative transition probabilities between pairs of brain states, hypnograms were downsampled to 10 s epochs. All laser stimulation trials were first aligned to the laser onset (t = 0 s). To calculate the cumulative probability that the animal transitions from state X at laser onset to state Y within **d** seconds, P(X→Y | t ≤ d), we first identified all **p** trials where the mouse was in state X at t = 0 s. Next, we counted the number of trials **q**, in which the mouse transitioned to state Y within **d** seconds. The cumulative probability was then calculated as P(X→Y | t ≤ **d**) = **p / q**. To quantify the maintenance of state X, we determined the cumulative probability P(X→X | t ≤ **d)**, i.e. the probability that, if the mouse was in state X at t = 0 s, it has not left state X within **d** seconds. For comparison, we repeated these computations for the 120 s interval preceding the laser onset and used the resulting values as baseline.

For statistical analysis, we performed a two-sided bootstrapping test. For each of the 10,000 bootstrap iterations, we resampled the complete data set (with **m** trials from **n** mice) by randomly selecting with replacement **m** laser stimulation trials from the **n** mice and then computing for each X→Y transition the cumulative transition probabilities for the 120 s baseline interval preceding the laser onset and the 120 s laser interval. Subtracting the areas under the curves (from 0 s to 120 s) for the baseline and laser interval probabilities yielded a sampling distribution of the paired mean difference between baseline and laser probabilities (the area ranging from 0 s to 120 s along the x-axis and 0 to 1 along the y-axis was normalized to 1), which was used to calculate CIs (ranging from the 2.5th to 97.5th percentile) and equal-tail bootstrap P-values^67^. In case all 10,000 sampled mean differences were consistently larger or smaller than 0, we set the P-value as P < 0.0001. The P-values were not corrected for multiple comparisons. To test whether the cumulative transition probabilities for transition X→Y significantly differed between experimental and control mice, we subtracted the sampled mean differences (between laser and baseline) for these two cohorts and used the resulting distribution to determine the CIs and P-values.

For representation, we visualized the relative changes in the cumulative transition probabilities between the baseline and laser interval using a directed graph, in which the color of each edge encodes the relative change in the probabilities between the laser and baseline interval (a relative change of 1 indicates no difference between baseline and laser). For each X→Y transition, the relative change was defined as the ratio of the areas under the laser and baseline curves.

### Spectral density and power estimation.

The power spectral density (PSD) of the EEG was computed using Welch’s method with Hanning window for consecutive 2 s, half-overlapping intervals. The PSD for a specific state (REM, NREM, wake, tonic REM or phasic θ event) was obtained by averaging across all epochs in the hypnogram labeled as that state. To compute the mean PSD for phasic θ events, we only included events with duration equal or larger than the 2 s Hanning window. To calculate the power within a given frequency band, we approximated the corresponding area under the PSD curve using a midpoint Riemann sum. To compute the EMG amplitude, we also first calculated the PSD of the EMG, integrated PSD values in the range 5 – 100 Hz, and then calculated the square root of the integral. To test the effects of laser stimulation during a specific brain state on the power within specific frequency bands of the PSD (or on the EMG), we determined for each mouse the δ (0.5 – 4.5 Hz), θ (6.0 – 9.5 Hz), and σ (10 – 15 Hz) power (or EMG amplitude) for that state with and without laser.

### Effect of laser on EEG spectrogram.

To determine time-dependent power bands, we first computed the EEG spectrogram using half-overlapping 5 s windows. To estimate within each 5 s window the PSD, we performed Welch’s method with Hanning window using sliding, half-overlapping 2 s intervals. Next, we normalized each frequency component in the spectrogram by its temporal mean across the entire recording (except for laser stimulation intervals), and then calculated for each frequency range and time point the corresponding Riemann sum. To test whether laser stimulation significantly changed the normalized EEG power within a given frequency band, we computed for each animal the mean power during the 120 s laser interval and the preceding 120 s baseline interval, and tested whether these values were significantly different across animals.

### Cross-correlation between ΔF/F activity and EEG power bands.

To calculate the cross-correlation between the ΔF/F signal of an imaged mPFC→LH cell and the σ or θ power, we first calculated for all NREMs bouts with duration ≥ 120 s (possibly interrupted by MAs) the σ power (**s**) from the EEG spectrogram using consecutive 2.5 s windows with 80% overlap to increase the temporal resolution. We normalized the spectrogram by dividing each frequency component by its mean power across the entire recording. Using the same overlapping time windows, we downsampled for each ROI the ΔF/F signal (**d**) and then calculated the cross-correlation of both signals. The cross-correlation was normalized by dividing it by the product of the standard deviation of **s** and **d** times the number of data points in s. To test whether the calcium activity within a subclass of cells was significantly correlated with a given power band, we first determined for each cell the value at the maximum (positive or negative) peak in the cross-correlation (in the range from −30 to 30 s time lag). We then tested using a one-sample t-test whether the peak values were significantly different from 0.

### Statistics and reproducibility.

No statistical methods were used to pre-determine sample sizes but our sample sizes are similar to those reported in previous publications^44,68^. We histologically verified that the location of fiber implants and virus expression were consistent across all animals in an experimental group. Representative histology images thus reflect the findings for all animals belonging to the same group. Statistical analyses were performed using the python modules scipy.stats (https://scipy.org), statsmodels (https://statsmodels.org), and pingouin (https://pingouin-stats.org). All statistical tests were two-sided and for all tests a P-value < 0.05 was considered as significant. The significance of changes in the cumulative transition probabilities between brain states induced by laser stimulation was tested using bootstrapping. Otherwise, data were compared using t-tests or using ANOVA followed by multiple comparisons tests (pairwise t-tests with Holm-Bonferroni correction for mixed or rm ANOVA, and Tukey’s post hoc test or Holm-Bonferroni correction for one-way ANOVA). Using the Shapiro-Wilk test, we verified that the data were normally distributed. For rm and mixed ANOVA, Mauchly’s test was applied to check the sphericity of the data. In case sphericity was violated, P-values were corrected using the Greenhouse-Geisser correction. Box plots were used to illustrate the distribution of data points. The upper and lower edges of the box correspond to the quartiles (25th and 75th percentile) of the dataset and the horizontal line in the box depicts the median, while the whiskers indicate the remaining distribution, except for outliers, i.e. points smaller than the 25th percentile - 1.5 * the interquartile range (IQR) or larger than the 75th percentile + 1.5 IQR. Outliers are depicted as diamonds. Statistical test results are included in the corresponding figure legends and related Supplementary tables.

## Data Availability

Large raw data files, including electrophysiological, video, or imaging data, are available upon request from the corresponding author. Source data for figures and Extended Data are supplied with this paper.
